# Inhibition of Angiopoietin-Like Protein 3 With a Monoclonal Antibody Reduces Triglycerides in Hypertriglyceridemia

**DOI:** 10.1161/CIRCULATIONAHA.118.039107

**Published:** 2019-06-27

**Authors:** Zahid Ahmad, Poulabi Banerjee, Sara Hamon, Kuo-Chen Chan, Aurelie Bouzelmat, William J. Sasiela, Robert Pordy, Scott Mellis, Hayes Dansky, Daniel A. Gipe, Richard L. Dunbar

**Affiliations:** 1 Division of Nutrition and Metabolic Diseases, Department of Internal Medicine, Center for Human Nutrition, University of Texas Southwestern Medical Center, Dallas (Z.A.).; 2 Regeneron Pharmaceuticals Inc, Tarrytown, NY (P.B., S.H., K.-C.C., A.B., W.JS., R.P., S.M., H.D., D.A.G.).; 3 Division of Translational Medicine and Human Genetics, Department of Medicine, Perelman School of Medicine at the University of Pennsylvania, Philadelphia (R.L.D.).

**Keywords:** cardiovascular disease, clinical trial, hypertriglyceridemia, lipids and lipoprotein metabolism

## Abstract

**Background:**

Hypertriglyceridemia is associated with increased cardiovascular risk and may be caused by impaired lipoprotein clearance. Angiopoietin-like protein 3 (ANGPTL3) inhibits lipoprotein lipase activity, increasing triglycerides and other lipids. Evinacumab, an ANGPTL3 inhibitor, reduced triglycerides in healthy human volunteers and in homozygous familial hypercholesterolemic individuals. Results from 2 Phase 1 studies in hypertriglyceridemic subjects are reported here.

**Methods:**

Subjects with triglycerides >150 but ≤450 mg/dL and low-density lipoprotein cholesterol ≥100 mg/dL (n=83 for single ascending dose study [SAD]; n=56 for multiple ascending dose study [MAD]) were randomized 3:1 to evinacumab:placebo. SAD subjects received evinacumab subcutaneously at 75/150/250 mg, or intravenously at 5/10/20 mg/kg, monitored up to day 126. MAD subjects received evinacumab subcutaneously at 150/300/450 mg once weekly, 300/450 mg every 2 weeks, or intravenously at 20 mg/kg once every 4 weeks up to day 56 with 6 months of follow-up. The primary outcomes were incidence and severity of treatment-emergent adverse events. Efficacy analyses included changes in triglycerides and other lipids over time.

**Results:**

In the SAD, 32 (51.6%) versus 9 (42.9%) subjects on evinacumab versus placebo reported treatment-emergent adverse events. In the MAD, 21 (67.7%) versus 9 (75.0%) subjects on subcutaneously evinacumab versus placebo and 6 (85.7%) versus 1 (50.0%) on intravenously evinacumab versus placebo reported treatment-emergent adverse events. No serious treatment-emergent adverse events or events leading to death or treatment discontinuation were reported. Elevations in alanine aminotransferase (7 [11.3%] SAD), aspartate aminotransferase (4 [6.5%] SAD), and creatinine phosphokinase (2 [3.2%) SAD, 1 [14.3%] MAD) were observed with evinacumab (none in the placebo groups), which were single elevations and were not dose-related. Dose-dependent reductions in triglycerides were observed in both studies, with maximum reduction of 76.9% at day 3 with 10 mg/kg intravenously (*P*<0.0001) in the SAD and of 83.1% at day 2 with 20 mg/kg intravenously once every 4 weeks (*P*=0.0003) in the MAD. Significant reductions in other lipids were observed with most evinacumab doses versus placebo.

**Conclusion:**

Evinacumab was well-tolerated in 2 Phase 1 studies. Lipid changes in hypertriglyceridemic subjects were similar to those observed with *ANGPTL3* loss-of-function mutations. Because the latter is associated with reduced cardiovascular risk, ANGPTL3 inhibition may improve clinical outcomes.

**Clinical Trial Registration:**

https://www.clinicaltrials.gov. Unique identifiers: NCT01749878 and NCT02107872.

## Abstract 2

Supplemental Digital Content is available in the text.

**Clinical Perspective**

**What Is New?**

- Low levels of triglycerides and other lipids are observed in individuals with loss-of-function mutations in *ANGPTL3*, an important regulator of lipid metabolism, providing a rationale for development of a monoclonal antibody therapy directed against this protein.
- The safety of evinacumab, a fully human ANGPTL3 antibody, was comparable with placebo, with no serious treatment-emergent adverse events, events related to death, or treatment discontinuation reported in 2 Phase 1 studies evaluating single and multiple ascending doses.
- Substantial and sustained percent reductions from baseline versus placebo were observed in triglycerides, with absolute levels reaching ~50 mg/dL for several evinacumab doses at specific time points in both studies.

**What Are the Clinical Implications?**

- The reduction in triglycerides and other lipid subfractions with ANGPTL3 inhibition by evinacumab is similar to the pan-hypolipidemia observed in individuals with loss-of-function mutations in *ANGPTL3*.
- The data from these 2 Phase 1 studies support further clinical evaluation of evinacumab in larger studies in hypertriglyceridemic individuals.
- Evinacumab could be a therapeutic option to lower elevated triglycerides and other lipids, especially in those with genetic mutations leading to hypertriglyceridemia or hypercholesterolemia with limited treatment options.

Elevated triglycerides or hypertriglyceridemia are associated with an increased risk of atherosclerotic cardiovascular disease.^1–3^ The National Lipid Association and National Cholesterol Education Program define borderline high hypertriglyceridemia when triglyceride levels are 150–199 mg/dL, high hypertriglyceridemia as 200–499 mg/dL, and very high or severe hypertriglyceridemia as ≥500 mg/dL.^4,5^ Severe hypertriglyceridemia is a well-established cause of acute pancreatitis.^6–9^

Hypertriglyceridemia may result from abnormalities in peripheral lipolysis or from overproduction or impaired clearance of lipoprotein.^10^ Chylomicrons and very-low-density lipoproteins (VLDLs) are the major triglyceride-rich lipoproteins synthesized in the intestine and liver, respectively.^11^ Hydrolysis of triglyceride-rich lipoproteins is mediated by lipoprotein lipase (LPL), the major enzyme acting on core triglycerides of chylomicrons and VLDL. Accordingly, carriers of loss-of-function (LOF) mutations in apolipoprotein C3, a protein that inhibits LPL activity, have lower levels of plasma triglycerides and are less prone to coronary heart disease.^3^ Additionally, variants of the LPL gene coding for LOF are associated with increased risk of coronary heart disease.^12^

Approved triglyceride-lowering drugs address triglyceride overproduction but have limited efficacy or are associated with adverse events and drug-drug interactions,^13^ indicating an urgent need for alternative therapeutic options for hypertriglyceridemic patients.^14–17^ Several outcome trials of triglyceride-lowering drugs failed to lower residual cardiovascular risk, notably fibrates, omega-3 fatty acids, and niacin.^18–23^ A recent cardiovascular outcomes study showed reduced residual risk with icosapent ethyl treatment in individuals with hypertriglyceridemia, on background statin therapy; the benefit observed was similar across baseline and attained triglyceride levels (≥150 or <150 mg/dL), indicating that the risk reduction observed may be partly attributable to metabolic effects other than triglyceride lowering.^24^

Angiopoietin-like protein 3 (ANGPTL3) is a secreted protein expressed primarily in the liver. It inhibits LPL activity and endothelial lipase activity, and retards clearance of triglyceride-rich lipoproteins upstream of low-density lipoprotein production.^25^ These actions raise plasma concentrations of triglycerides and high-density lipoprotein cholesterol (HDL-C).

Conversely, LOF mutations in *ANGPTL3* lead to ANGPTL3 deficiency and pan-hypolipoproteinemia.^26–28^ Thus, individuals with such mutations have lower plasma triglycerides, low-density lipoprotein cholesterol (LDL-C), and HDL-C.^25,27,29–32^ A genetic study of a family with hypobetalipoproteinemia found that the nonsense mutations in *ANGPTL3* affected phenotypes in a manner dependent on the gene dosage: those heterozygous with 1 of the 2 nonsense mutations had significantly lower plasma levels of LDL-C (72 mg/dL [1.9 mmol/L]) and triglycerides (64 mg/dL [0.7 mmol/L]) compared with those with neither mutation (LDL-C, 109 mg/dL [2.8 mmol/L]; *P*<0.001; triglycerides, 130 mg/dL [1.5 mmol/L]; *P*=0.01); compound heterozygotes had even lower plasma LDL-C (33 mg/dL [0.9 mmol/L]) and triglyceride levels (21 mg/dL [0.2 mmol/L])^25^ No adverse sequelae, such as liver disease associated with other causes of low cholesterol,^33^ have been reported.

Evinacumab is a fully human monoclonal antibody generated using VelocImmune technology,^34,35^ and is a specific inhibitor of ANGPTL3 that reduces triglycerides, non–HDL-C, and LDL-C in healthy human volunteers and in patients with homozygous familial hypercholesterolemia.^36,37^ In this study, we report the full safety and efficacy data from single and multiple ascending dose Phase 1 studies of evinacumab in subjects with mild to moderately elevated triglycerides and/or LDL-C.

## Methods

Qualified researchers may request access to study documents (including the clinical study report, study protocol with any amendments, blank case report form, and statistical analysis plan) that support the methods and findings reported in this manuscript. Individual anonymized participant data will be considered for sharing once the indication has been approved by a regulatory body, if there is legal authority to share the data and there is not a reasonable likelihood of participant reidentification. Submit requests to https://errs.regeneron.com/external. The study protocols were approved by the investigational review board at each study center, and all subjects provided written informed consent.

### Study Designs

We performed two separate Phase 1 clinical studies of evinacumab. The first was a first-in-human, randomized, single ascending dose, placebo-controlled, double-blind study of the safety, tolerability, and bioeffect of evinacumab administered subcutaneously (SC) or intravenously (IV) in otherwise healthy men and women with mixed dyslipidemia (defined as elevated triglycerides [150 to 450 mg/dL] or LDL-C [≥100 mg/dL]; trial identifier, NCT01749878). The study also included groups enrolling individuals with triglycerides ≥450 mg/dL or ≥1000 mg/dL, whose results will be reported in a separate manuscript. The second Phase 1 study was a randomized, double-blind, placebo-controlled, multiple ascending dose study of the safety and tolerability of evinacumab in otherwise healthy men and women with elevated triglycerides (150 to 500 mg/dL inclusive) and LDL-C (≥100 mg/dL; trial identifier, NCT02107872).

### Single Ascending Dose Study

Subjects were enrolled at 3 sites in the United States. The cohort comprised otherwise healthy men and women aged 18 to 65 years with LDL-C ≥100 mg/dL (directly measured) at screening and/or serum triglycerides ≥150 and ≤450 mg/dL (measured after at least a 12-hour fast). The full study criteria are detailed in Table I in the online-only Data Supplement.

Sterile, lyophilized evinacumab (50 mg/mL) was supplied in a 5-mL single-use glass vial with a 2.0-mL withdrawable volume. Placebo was supplied in matched vials. The study drug was administered at baseline (day 1) by study personnel. All SC drug administrations were given as 1 or more injections into the abdominal area.

The study involved 6 sequential ascending-dose levels: 3 SC (75, 150, and 250 mg) and 3 IV (5, 10, and 20 mg/kg). Both SC and IV dose levels were divided into 2 cohorts based on the subjects’ lipid parameters at screening, for a total of 12 cohorts. Subjects were randomized at a ratio of 3:1 (evinacumab:placebo); within each dose level, a maximum of 12 subjects received evinacumab and 4 received placebo.

The study consisted of a screening period, a baseline visit, and a treatment and observation period. Subjects underwent screening for study eligibility from day −21 to day −2, and eligible subjects were admitted to the clinic on day −1 for predose study procedures. Subjects were randomized to receive evinacumab or placebo on day 1 (baseline). They remained in the clinic until day 4 assessments had been completed, and returned to the clinic for outpatient visits and laboratory evaluations. For the first 4 dose levels (75, 150, and 250 mg SC and 5 mg/kg IV), the outpatient visits and laboratory evaluations were on days 8, 11, 15, 22, 29, 43, 64, 85, and 106 (end of study). For the 5th and 6th dose levels (10 and 20 mg/kg IV), the outpatient visits and laboratory evaluations were on days (±2 days) 8, 11, 15, 22, 29, 43, 64, 85, and 126 (end of study).

### Multiple Ascending Dose Study

The study population comprised otherwise healthy men and women aged between 18 to 65 years with elevations in both triglycerides (150 to 500 mg/dL, measured after at least an 8-hour fast) and LDL-C (≥100 mg/dL) at screening, enrolled at 6 sites in the United States. The full study criteria are detailed in Table II in the online-only Data Supplement.

The study population was divided into 6 cohorts of evinacumab dose level and regimen, namely cohort 1 at 150 mg SC once every week (QW); cohort 2 at 300 mg SC once every 2 weeks (Q2W); cohort 3 at 300 mg SC QW; cohort 4 at 450 mg SC Q2W; cohort 5 at 450 mg SC QW; and cohort 6 at 20 mg/kg IV once every 4 weeks (Q4W). Within each cohort, 8 subjects were randomized at an evinacumab:placebo ratio of 3:1. The study consisted of 3 periods: screening, double-blind treatment, and follow-up. Subjects underwent screening for study eligibility from day −21 to day −2. On study day −1, eligible subjects were admitted to the clinic for predose study procedures. Subjects were randomized and started to receive study drug on day 1 (baseline). Subjects remained in the clinic until day 3 assessments had been completed, and returned to the clinic for outpatient visits and laboratory evaluations on days 8, 15, 22, 29, 36, 43, 50, 57, 78, 99, 120, 141, 162, and 183 (end of study); study treatment ended on day 50 for cohorts 1, 3, and 5, on day 43 for cohorts 2 and 4, and on day 29 for cohort 6. The outpatient visits occurred within a window of ±2 days.

### Outcomes Assessment: Single and Multiple Ascending Dose Studies

Safety and tolerability were assessed by physical examination, vital signs, electrocardiogram data, and clinical evaluation. Subjects were asked to monitor all adverse events experienced from the time of informed consent until their end-of-study visit. Acute administration reactions were defined as any adverse event that occurred during the study drug administration or within 2 hours postdose. Elevations in alanine aminotransferase, aspartate aminotransferase, and creatine phosphokinase reported as adverse events were based on the reporting physicians’ judgment.

In both studies, pharmacodynamic effects were assessed by analysis of lipids over time, and included triglycerides, VLDL cholesterol (VLDL-C), non-HDL-C, apolipoprotein B, LDL-C, lipoprotein(a) (Lp[a]), HDL-C, apolipoprotein A1, and total cholesterol.

### Statistical Analysis

The primary outcome for both studies was the incidence and severity of treatment-emergent adverse events (TEAEs), reported from the first dose of study drug to the end of study, or the last visit in subjects treated with evinacumab or placebo. No formal statistical hypotheses were planned for the studies. All *P* values presented in this article are nominal based on an analysis of covariance model with the baseline value as covariate for all lipid parameters except triglycerides and Lp(a). A rank-base analysis of covariance model was used for triglycerides and Lp(a). The safety analysis set included all randomized subjects exposed to at least part of 1 dose of study treatment; subjects were analyzed based on the treatment received (intent-to-treat). Treatment compliance/administration and all clinical safety variables were analyzed using the safety analysis set.

Sample size estimation and dose-escalation and stopping rules are detailed in Text I and II in the online-only Data Supplement. For continuous variables, descriptive statistics included the number of subjects reflected in the calculation (n), mean, median, standard deviation (SD), minimum, and maximum. For categorical or ordinal data, frequencies and percentages are displayed for each category. Formal comparisons and testing were not planned against the placebo cohort or between the dose levels. Safety and tolerability are summarized descriptively by dose level and cohort, and included TEAEs, laboratory variables, and vital signs. Placebo-treated subjects in the different dose groups were pooled into a single cohort.

## Results

### Subjects

#### Single Ascending Dose Study

Of 83 enrolled subjects, 62 were randomized to single ascending dose evinacumab and 21 to placebo (Figure I in the online-only Data Supplement). All 83 (100%) randomized subjects received study drug and were included in the safety analysis. Seventy (84.3%) completed the study; of those who did not, 8 (9.6%) withdrew consent and 5 (6.0%) were lost to follow-up. Baseline characteristics and lipid profile are detailed in Table 1. Dose-dependent increases in total ANGPTL3 concentrations with maximum levels at day 4 were observed after administration of both SC and IV evinacumab, indicating target binding (Figure IIA in the online-only Data Supplement). Mean maximum concentrations increased from 0.11 mg/L to 0.56 mg/L as dose increased from 75 mg SC to 20 mg/kg IV. The pharmacokinetic parameters of evinacumab serum levels are presented in Table III in the online-only Data Supplement.

**Table 1.**
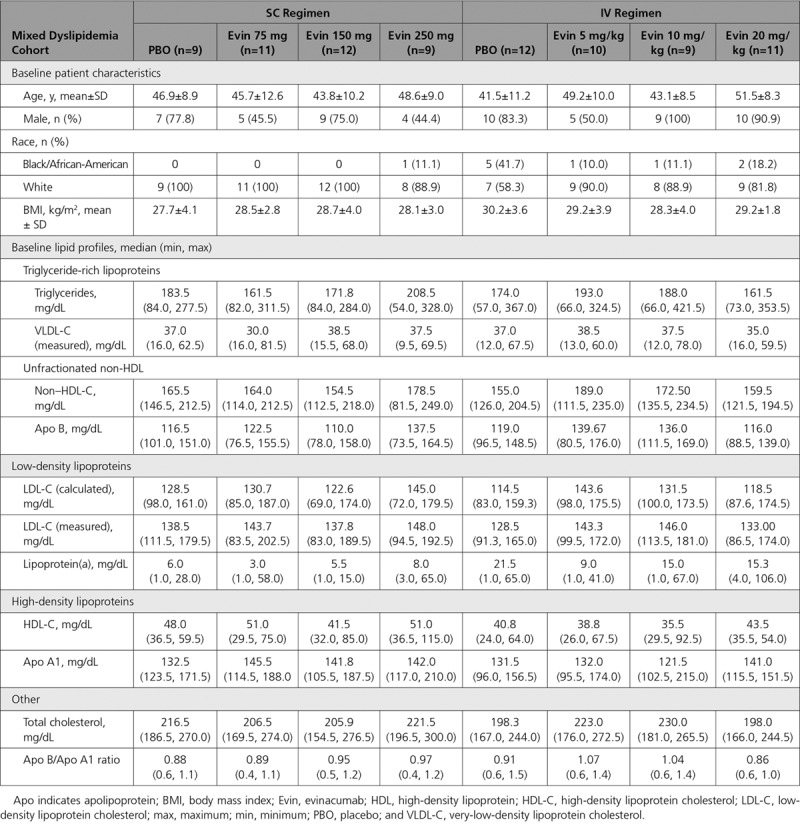
Baseline Characteristics and Lipid Profile of Subjects With Mixed Dyslipidemia (Single Ascending Dose Study)

#### Multiple Ascending Dose Study

Fifty-six subjects (46 in the SC regimen, 10 in the IV regimen) were randomized in the multiple ascending dose study, of whom 52 (43 SC, 9 IV) received study treatment (Figure IB in the online-only Data Supplement). The 4 randomized but not treated subjects were in the following treatment groups (1 subject each): placebo, evinacumab 300 mg SC Q2W, 450 mg SC Q2W, and 20 mg/kg IV Q4W. In each case, the investigator decided to forgo study treatment before the first dose as they did not meet study criteria after randomization. Thirty-nine (84.8%) subjects allocated to the SC regimen and 8 (80.0%) to the IV regimen completed the study. Nine subjects withdrew prematurely from the study: 7 (15.2%) SC and 2 (20.0%) IV. The primary reasons for study discontinuation were investigator/sponsor decision and withdrawal of consent. The subjects’ baseline characteristics and lipid profile are detailed in Table 2. Baseline total ANGPTL3 concentrations were similar across different evinacumab treatment groups, with the mean ranging from 0.09 to 0.11 mg/L. After evinacumab administration, a dose-dependent increase in total ANGPTL3 from baseline was observed in all groups (Figure IIB in the online-only Data Supplement; pharmacokinetics of evinacumab serum levels presented in Table III in the online-only Data Supplement). The maximum ANGPTL3 serum concentration of ~0.6 mg/L occurred on day 29, after the second dose in 20 mg/kg IV Q4W group. As with the single-dose study, the changes in ANGPTL3 levels observed indicated target binding of evinacumab with ANGPTL3 at all doses tested.

**Table 2.**
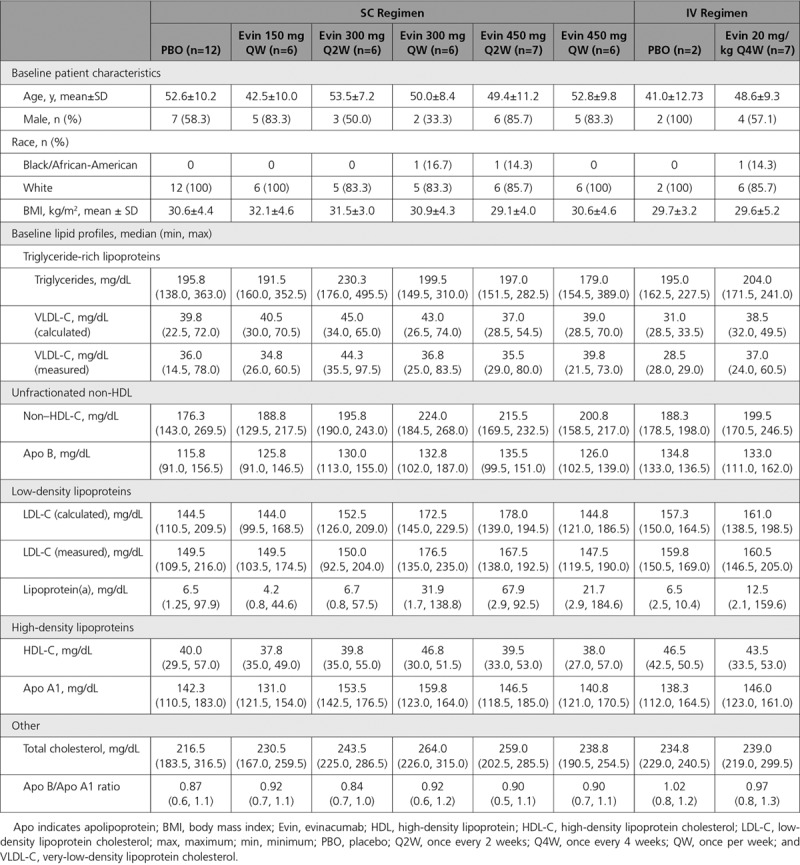
Baseline Characteristics and Lipid Profile of Subjects With Elevated Triglycerides and LDL-C (Multiple Ascending Dose Study)

### Safety and Tolerability

#### Single Ascending Dose Study

No dose-limiting toxicities were observed in the single ascending dose study. The maximum dose of evinacumab evaluated was 20 mg/kg IV. Thirty-two of 62 (51.6%) subjects in the evinacumab group and 9 of 21 (42.9%) in the placebo group experienced at least 1 TEAE (Table 3). No events met the criteria for a serious TEAE or led to death or treatment discontinuation. Fifteen (24.2%) subjects in the evinacumab group and 1 (4.8%) in the placebo group experienced a TEAE that was considered related to the study drug. Seven subjects in the evinacumab group (11.3% versus 0% in the placebo group) had increased alanine aminotransferase levels reported as adverse events based on reporting physicians’ judgment: 2 with elevated levels (defined by laboratory tests as >3 times upper limit of normal [ULN] and baseline ≤5 times ULN) and 5 with <3 times ULN. Four subjects (6.5% on evinacumab versus 0% on placebo) had increased aspartate aminotransferase (<3 times ULN). Two subjects on evinacumab (3.2% versus 0% on placebo) reported increased creatine phosphokinase levels as adverse events based on reporting physicians’ judgment. Based on laboratory evaluation, 6 (9.7%) subjects on evinacumab (versus 3 [14.3%] on placebo) had elevated creatine kinase (>3 times ULN and baseline creatine phosphokinase ≤3 times ULN). No subject experienced an elevation of total bilirubin >1.5 times ULN. No subjects met the criteria for Hy’s law. Changes over time in liver function test parameters are shown in Tables IV through VI in the online-only Data Supplement.

**Table 3.**
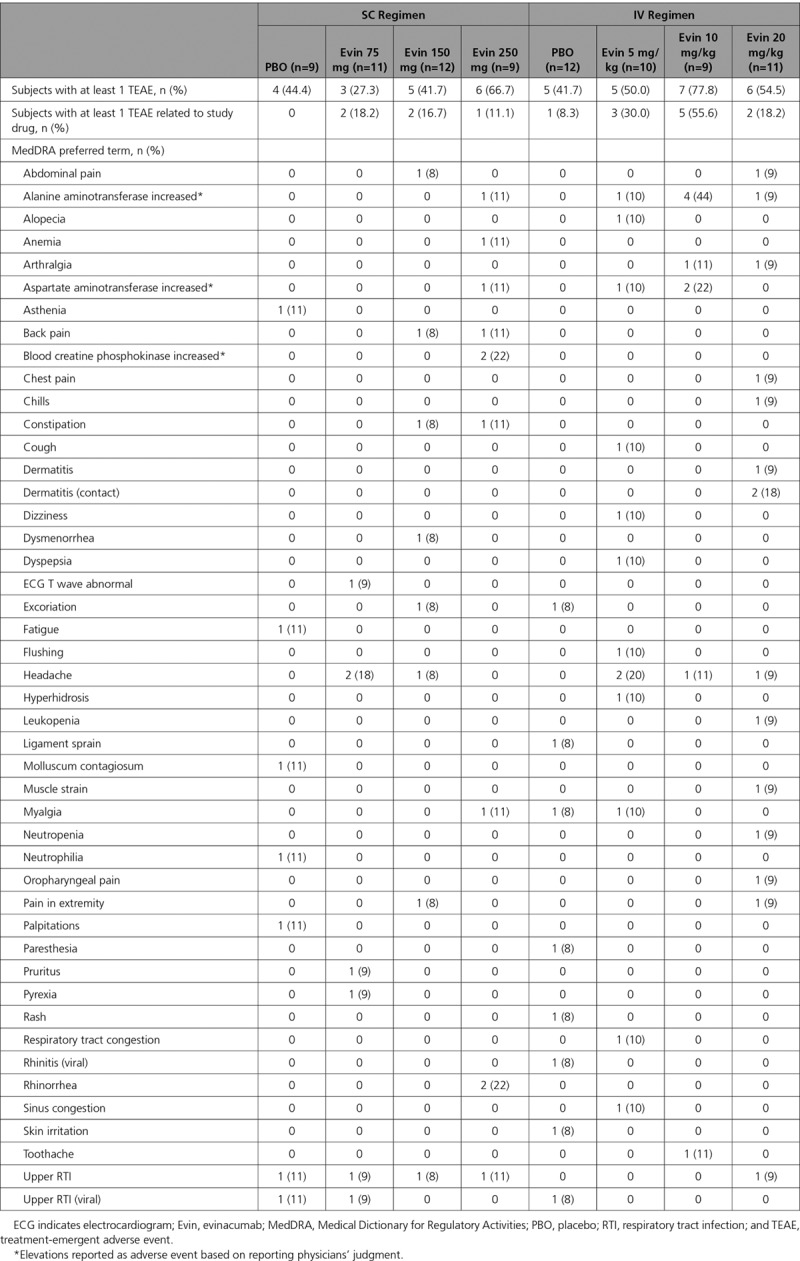
Treatment-Emergent Adverse Events Overall and by MedDRA Preferred Term in Subjects With Mixed Dyslipidemia (Single Ascending Dose Study)

#### Multiple Ascending Dose Study

Thirty subjects (69.8%) on the multiple ascending dose SC regimen reported at least 1 TEAE: 21 (67.7%) with evinacumab and 9 (75.0%) with placebo (Table 4). Overall, 6 subjects in the 450 mg QW group, 3 in the 150 mg QW group, and 5 each in the 300 mg Q2W and 300 mg QW groups experienced TEAEs. There were more drug-related TEAEs with evinacumab (35.5%) than placebo (16.7%). Specific events occurred sporadically with no pattern detected; the most commonly reported was headache (reported in 3 [25.0%] patients receiving placebo, 3 [50.0%] patients receiving evinacumab 300 mg Q2W, and 2 [33.3%] patients receiving evinacumab 450 mg QW). There were no serious TEAEs or deaths during the study, and none of the subjects discontinued treatment because of a TEAE. With the IV regimen, 7 (77.8%) subjects reported at least 1 TEAE: 6 (85.7%) in the evinacumab group and 1 (50.0%) in the placebo group (Table 5). Two (22.2%) subjects in the evinacumab group reported drug-related TEAEs. None of the events were serious, and no subjects died or discontinued treatment. Elevations in alanine aminotransferase and aspartate aminotransferase were reported as adverse events in 1 subject (8.3% for both) in the placebo SC group (none in evinacumab SC and IV groups). Increased creatine phosphokinase was reported as an adverse event in 1 subject (14.3%) treated with evinacumab IV (none with placebo). Changes over time in liver function test parameters are shown in Tables VII through IX in the online-only Data Supplement.

**Table 4.**
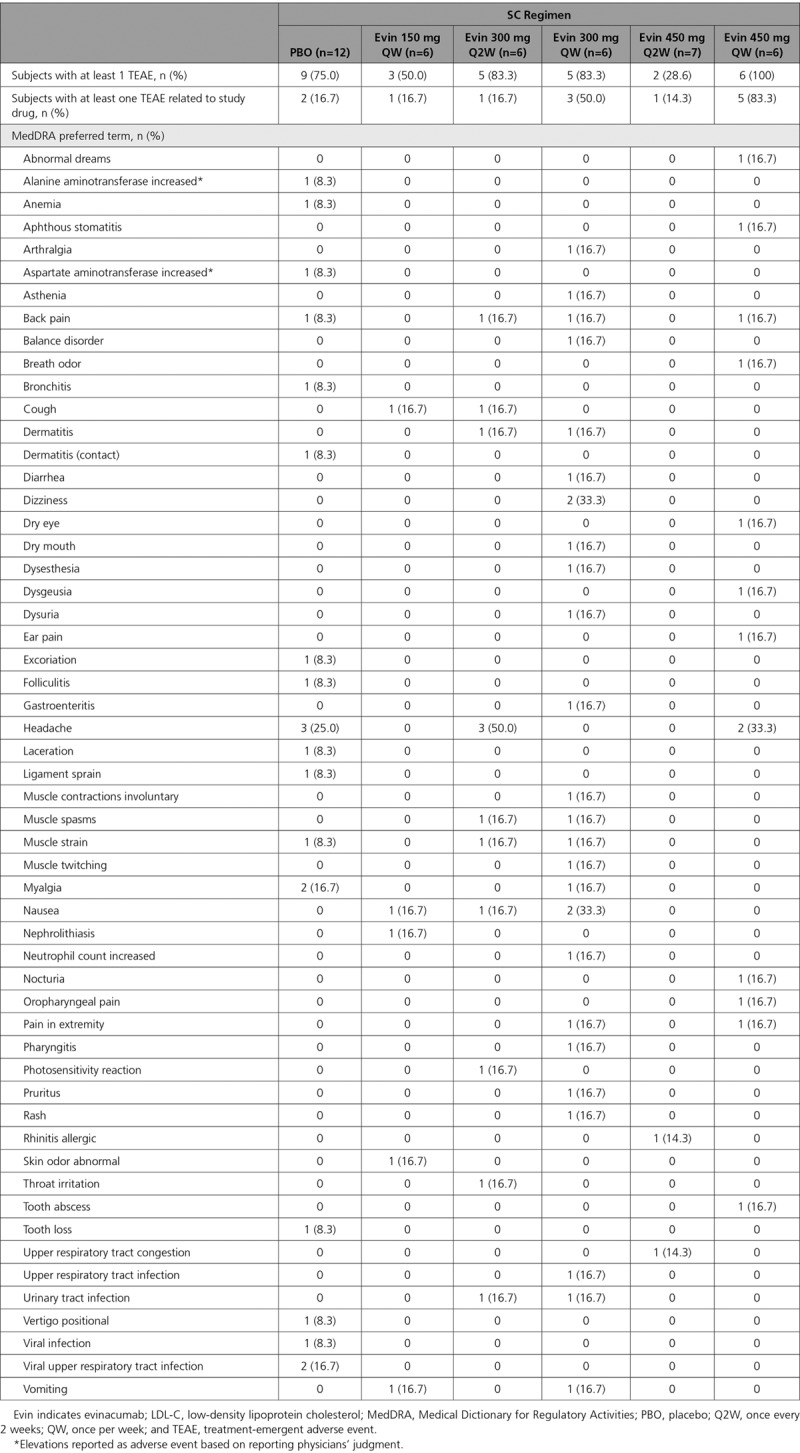
Treatment-Emergent Adverse Events Overall and by MedDRA Preferred Term in Subjects With Elevated Triglycerides and LDL-C (Multiple Ascending Dose Study, SC doses)

**Table 5.**
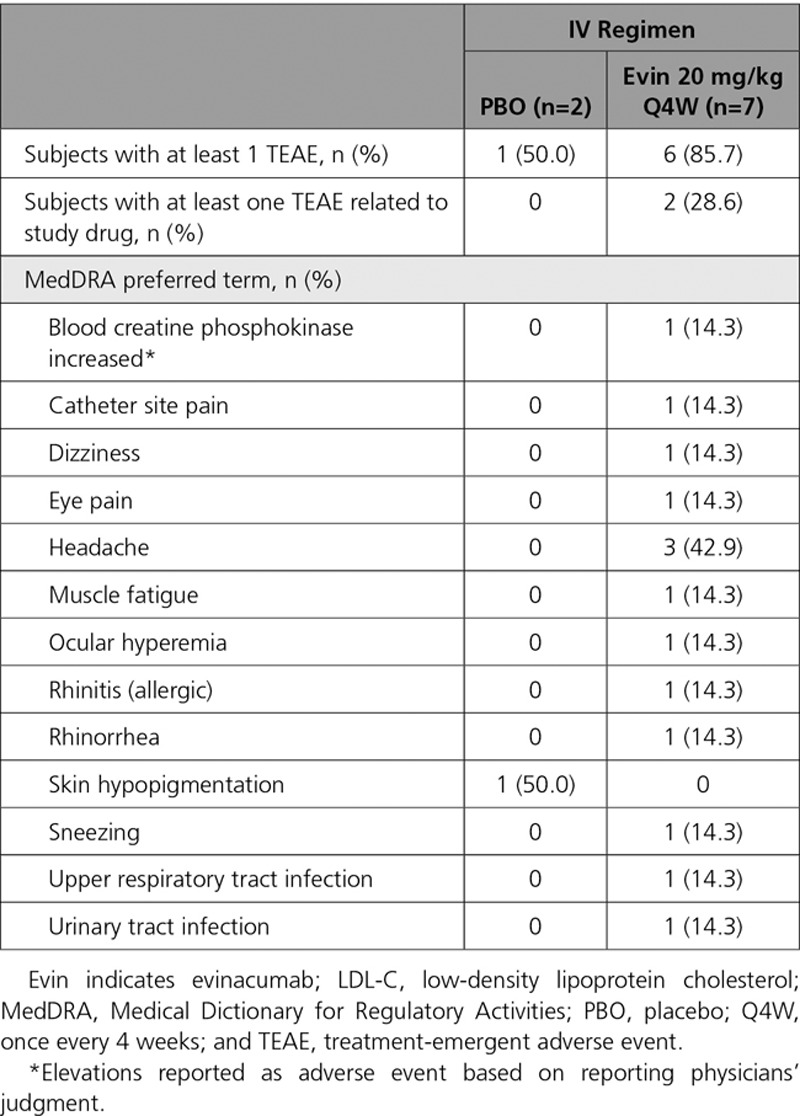
Treatment-Emergent Adverse Events Overall and by MedDRA Preferred Term in Subjects With Elevated Triglycerides and LDL-C (Multiple Ascending Dose Study, IV doses)

### Triglyceride-Rich Lipoproteins

#### Single Ascending Dose Study

**Figure 1.**
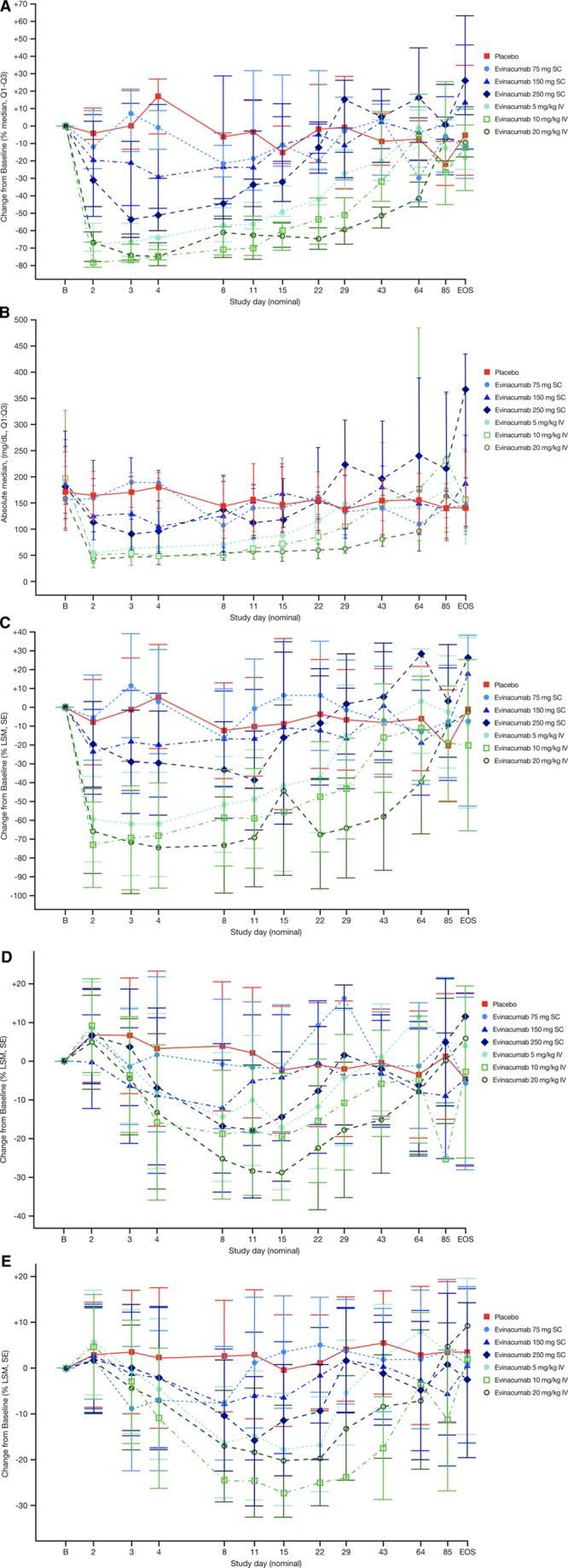
**Percent median (Q1–Q3) change from baseline**. Percent median (Q1–Q3) change from baseline in (**A**) triglycerides, (**B**) absolute median triglyceride levels, and percent LSM (SE) change from baseline in (**C**) VLDL-C, (**D**) LDL-C, and (**E**) HDL-C in subjects with multiple dyslipidemia (single ascending dose study). EOS indicates end of study; HDL-C, high-density lipoprotein cholesterol; LDL-C, low-density lipoprotein cholesterol; LSM, least-squares mean; and VLDL-C, very-low-density lipoprotein cholesterol.

A single SC dose of evinacumab reduced triglycerides dose-dependently, with the greatest median reduction versus placebo from baseline at day 4 provided by the 250-mg dose (−55.5%, *P*<0.0001; Figure 1A). Evinacumab IV reduced triglyceride levels more profoundly by day 4: median difference versus placebo of −80.3% with 5 mg/kg (*P*<0.0001), −88.0% with 10 mg/kg (*P*<0.0001), and –83.9% with 20 mg/kg (*P*<0.0001; Figure 1A). Triglycerides were reduced to ~50 mg/dL on day 2, and these reductions were sustained up to day 11 with several of the doses of evinacumab tested (Figure 1B). The absolute triglyceride level seemed to floor at ~50 mg/dL with a single dose of evinacumab at 5 mg/kg IV and did not reduce further with higher doses (10 and 20 mg/kg IV). Higher doses of evinacumab did, however, progressively extend the duration of maximum triglyceride reduction. VLDL-C levels decreased on day 4 after evinacumab administration, with a more profound least-squares mean (LSM) difference versus placebo via IV than via SC (Figure 1C): −67.9% with 5 mg/kg IV (*P*<0.0001), −73.8% with 10 mg/kg IV (*P*<0.0001), and −80.1% with 20 mg/kg IV (*P*<0.0001). A floor effect was not observed for VLDL with the evinacumab doses tested.

#### Multiple Ascending Dose Study

**Figure 2.**
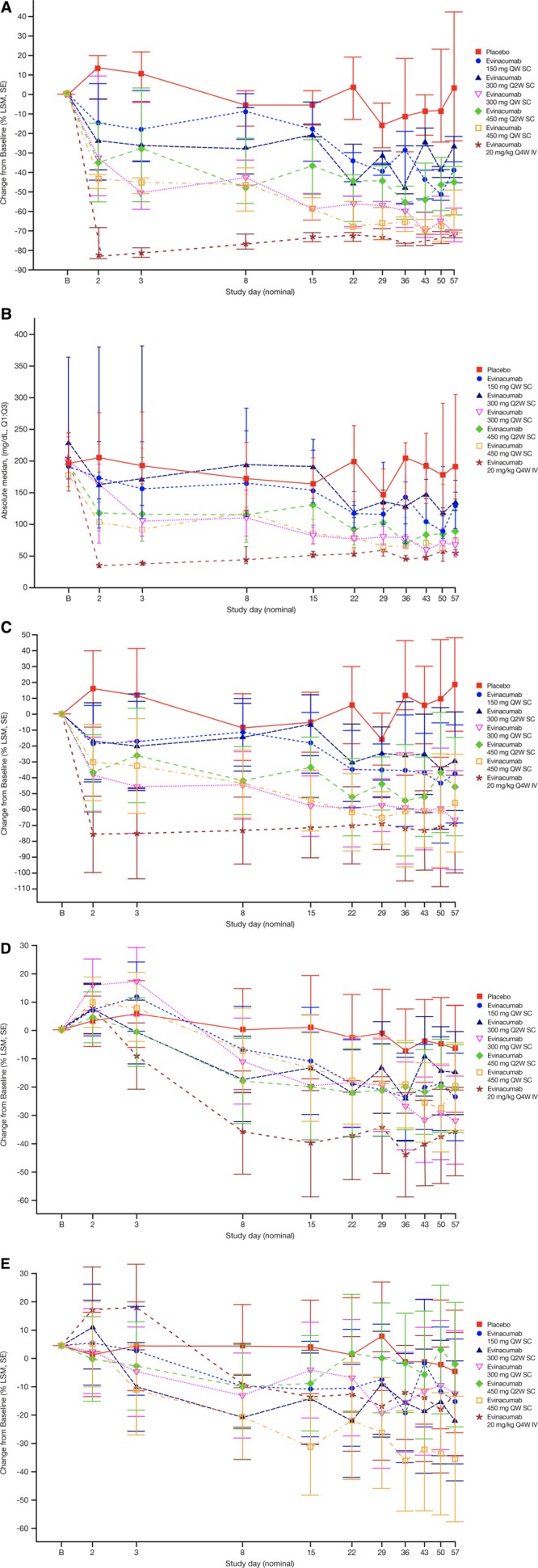
**Percent median (Q1–Q3) change from baseline**. Percent median (Q1–Q3) change from baseline in (**A**) triglycerides, (**B**) absolute median triglyceride levels, and percent LSM (SE) change from baseline in (**C**) VLDL-C, (**D**) LDL-C, and (**E**) HDL-C in subjects with elevated triglycerides and LDL-C (multiple ascending dose study). HDL-C indicates high-density lipoprotein cholesterol; LDL-C, low-density lipoprotein cholesterol; LSM, least-squares mean; Q2W, every 2 weeks; Q4W, once every 4 weeks; QW, once per week; and VLDL-C, very low-density lipoprotein cholesterol.

Triglycerides decreased in all multiple ascending dose evinacumab groups and increased in the placebo group. Evinacumab treatment resulted in median change versus placebo of −88.2% (*P*=0.0003) in the 20-mg/kg IV group beginning on day 2. Median differences versus placebo of −51.9% (*P*=0.0006) and −50.3% (*P*=0.0012) were observed in the 300 mg QW and 450 mg QW SC dose groups, respectively, beginning on day 15 (Figure 2A). These reductions in triglycerides were sustained in the higher SC dose groups through day 57, and in the 20-mg/kg IV dose group through day 99. Reduction of triglycerides to ~50 mg/dL was also observed with the evinacumab IV 20 kg/mg dose regimen Q4W (Figure 2B). Similarly, calculated VLDL-C decreased from baseline in all evinacumab groups (Figure 2C). LSM differences versus placebo in VLDL-C of −53.9% (*P*<0.0001) with 300 mg QW SC, −46.1% (*P*=0.0003) with 450 mg QW SC, and −91.2% (*P*<0.0001) with 20 mg/kg IV were observed on day 2 and sustained at greater than −70.0% through day 57. The mean difference versus placebo in VLDL-C on day 57 ranged from −48.1% to −64.0% in the other SC groups.

### Unfractionated Non–High-Density Lipoproteins

#### Single Ascending Dose Study

Unfractionated non–HDL-C levels decreased after evinacumab administration in the single ascending dose study, but this reduction did not occur as rapidly as triglycerides. Non–HDL-C fell to minimum levels at day 11: LSM difference versus placebo were –31.2% with 10 mg/kg IV, and –35.4% with 20 mg/kg IV (both *P*<0.0001; Figure IIIA in the online-only Data Supplement). The effect on non–HDL-C levels was more profound with the IV than the SC regimen. A maximum LSM difference versus placebo of –23.7% (*P*<0.0001) was observed with 250 mg at day 11 among the SC doses tested. Likewise, apolipoprotein B levels decreased versus placebo (LSM difference) by –21.4% with 10 mg/kg IV evinacumab and –26.8% with 20 mg/kg evinacumab IV by day 11 (both *P*<0.0005; Figure IIIB in the online-only Data Supplement). Absolute levels of apolipoprotein B were reduced over time with evinacumab (Figure IIIC in the online-only Data Supplement).

#### Multiple Ascending Dose Study

Non–HDL-C decreased from baseline to day 57 in all multiple ascending dose groups, with the maximum mean percentage reduction in the 20-mg/kg IV dose group on day 36 (LSM difference versus placebo of −45.8%, *P*<0.0001; Figure IVA in the online-only Data Supplement). On day 57, non–HDL-C fell versus placebo: −39.8% (LSM) with evinacumab 20 mg/kg IV (*P*<0.0001), compared with −24.5% with 150 mg QW (*P*=0.0004), −14.7% with 300 mg Q2W (*P*=0.0351), −37.5% with 300 mg QW (*P*<0.0001), −22.6% with 450 mg Q2W (*P*=0.0005), and −26.2% with 450 mg QW SC (*P*=0.0002); these drops were not dose-related. Similarly, apolipoprotein B fell from baseline in all evinacumab groups; the LSM difference versus placebo on day 57 was −30.7% on 20 mg/kg IV (*P*<0.0001), compared with −13.0% to −23.7% in the SC groups (Figure IVB in the online-only Data Supplement). Absolute levels of apolipoprotein B over time are shown in Figure IVC in the online-only Data Supplement.

### Low-Density Lipoproteins

#### Single Ascending Dose Study

In subjects treated with evinacumab 150 mg SC, a statistically significant reduction in LDL-C levels (LSM difference versus placebo) was observed at day 3 (−12.9%, *P*=0.0198) and at day 8 (−15.9%, *P*=0.0100). On evinacumab 250 mg SC, LDL-C fell significantly on day 8 (−20.6%, *P*=0.0072) through day 11 (−20.3%, *P*=0.0091). A statistically significant drop in LDL-C levels occurred with 5 mg/kg IV on day 8 (−18.2%, *P*=0.0057); with 10 mg/kg IV on day 4 (−19.0%, *P*=0.0197), day 8 (−22.8%, *P*=0.0010), and through day 22 (−14.5%, *P*=0.0349); and with 20 mg/kg IV from day 4 (−16.3%, *P*=0.0321) to day 43 (−14.8%, *P*=0.0079; Figure 1D).

#### Multiple Ascending Dose Study

LDL-C fell from baseline in all evinacumab groups (Figure 2D); the maximum LSM differences versus placebo of −22.0% (*P*=0.0194) and −25.1% (*P*=0.0074) on day 57 were seen with 300 mg QW SC and 20 mg/kg IV, respectively.

In both studies, Lp(a) showed no dose-dependent effect across the evinacumab treatment groups (Figures VA and VIA in the online-only Data Supplement).

### High-Density Lipoproteins

#### Single Ascending Dose Study

Evinacumab treatment resulted in statistically significant reductions versus placebo in HDL-C in all treatment groups by day 8 (Figure 1E). At day 8, the LSM differences versus placebo with evinacumab 75 mg SC, 150 mg SC, and 250 mg SC were −10.3% (*P*=0.0394), −10.5% (*P*=0.0194), and −12.9% (*P*=0.0174), respectively. On IV evinacumab, the LSM difference versus placebo at day 8 was −18.8% at 5 mg/kg, −27.3% at 10 mg/kg, and −19.6% at 20 mg/kg (all *P*≤0.0001). The maximum mean percent HDL-C reductions versus placebo were seen with evinacumab treatment at 250 mg/kg SC (−19.0%, day 11) and with 10 mg/kg IV (−28.2%, day 29). Apart from the 20 mg/kg IV treatment group, the duration of HDL-C reduction was dose-dependent, and HDL-C appeared to remain suppressed until the end of the study. In all evinacumab groups, apolipoprotein A1 reductions resembled those of HDL-C, with the maximum LSM percent drop versus placebo observed in the 10-mg/kg IV dose group (−29.8%, day 15; Figure VB in the online-only Data Supplement).

#### Multiple Ascending Dose Study

HDL-C fell from baseline in most of the evinacumab groups (Figure 2E). At day 57, the percent change from baseline (LSM difference versus placebo) with evinacumab SC at 150 mg QW was −8.2% (*P*=0.3327), 300 mg Q2W was −13.5% (*P*=0.1270), 300 mg QW was −6.0% (*P*=0.4756), 450 mg Q2W was +1.9% (*P*=0.8081), and 450 mg QW was −23.9% (*P*=0.0068). The LSM difference versus placebo with evinacumab IV at 20 mg/kg Q4W was −6.2% (*P*=0.4585). Apolipoprotein A1 fell in all evinacumab groups, with the maximum percent change from baseline (LSM difference versus placebo) observed on day 57 at −37.7% (*P*<0.0001) in the 450-mg QW SC group; in the other SC groups, percent changes ranged from −12.3% to −21.5%. Treatment with evinacumab 20 mg/kg IV resulted in percent change from baseline (LSM difference versus placebo) of −22.0% (*P*<0.0001; Figure VIB in the online-only Data Supplement).

### Total Cholesterol

#### Single Ascending Dose Study

Mean percent change in total cholesterol decreased from baseline rapidly up to day 15 in all evinacumab groups; maximum LSM differences versus placebo were seen on day 11 in the 10 mg/kg and 20 mg/kg IV dose groups at −30.3% and −32.4%, respectively (*P*<0.0001 for both; Figure VC in the online-only Data Supplement).

#### Multiple Ascending Dose Study

Mean percent change in total cholesterol decreased from baseline beginning on day 8 through day 57 in all evinacumab groups. At day 57, maximum LSM differences versus placebo of −33.8% and −32.4% were seen in the 20 mg/kg IV (*P*<0.0001) and 300 mg QW SC (*P*<0.0001) groups, respectively (Figure VIC in the online-only Data Supplement).

## Discussion

Treatment with the fully human monoclonal ANGPTL3-blocking antibody evinacumab of individuals with mixed dyslipidemia with borderline high triglycerides prompted substantial, sustained reductions in triglycerides and VLDL-C levels in 2 randomized, double-blind, placebo-controlled, Phase 1 studies. In the single ascending dose study, triglyceride suppression was dose-dependent and rapid, with maximum drops at day 3. The magnitude of triglyceride reduction observed with evinacumab was dose-dependent up to a dose of 5 mg/kg IV. The higher doses of 10 and 20 mg/kg IV extended the duration of triglyceride suppression but did not reduce the minimum level of triglycerides attained (~50 mg/dL). The highest dose of evinacumab lowered triglycerides by ~80%; in general, reductions in triglycerides were more profound on IV versus SC dosing. In the multiple ascending dose study, median reductions in triglycerides at day 57 were ~70% for each of the 300 mg QW SC, 450 mg QW SC, and 20 mg/kg Q4W IV doses of evinacumab.

There is a persistent and ongoing medical need for new drugs to lower triglycerides in patients with hypertriglyceridemia.^38^ Evinacumab could be an option to help with this gap as an adjunct or alternative therapy for patients with otherwise irremediable levels of triglycerides or LDL-C. Significant reductions were also observed in non–HDL-C, apolipoprotein B, total cholesterol, HDL-C, and apolipoprotein A1 levels in most evinacumab treatment groups compared with placebo. ANGPTL3 inhibition increases activity of endothelial lipase and, therefore, decreases HDL-C levels.^39^ Although the inactivation of ANGPTL3 by evinacumab increases activity of LPL in humans and animal models,^40–42^ the exact mechanisms by which LDL-C and other lipoproteins are reduced by ANGPTL3 inhibition are unknown. Evinacumab treatment decreased LDL-C and HDL-C levels, which may contribute to the reduced levels of total cholesterol observed in both studies. Evinacumab treatment in both studies did not result in significant changes in Lp(a) levels. Our data of ANGPTL3 inhibition with evinacumab evoke previous observations in patient populations where LOF mutations in *ANGPTL3* led to pan-hypolipidemia.^26–28^ Results from the single ascending dose study of evinacumab treatment of patients with severe hypertriglyceridemia and *LPL* pathway mutations are being reported in a separate publication.

Evinacumab treatment reduced LDL-C levels in both studies, with maximum LDL-C reductions versus placebo observed between day 11 and day 15 in a dose-dependent manner in the single ascending dose study, and at day 57 in the 300-mg QW SC and 20 mg/kg IV groups in the multiple ascending dose study. Contrary to the expected increase in LDL-C attributable to LPL activation, we observed a reduction in LDL-C with ANGPTL3 inhibition by evinacumab. Again, the mechanism whereby ANGPTL3 inhibition reduces LDL-C levels remains elusive but is known to be independent of its effects on triglycerides and LDL receptor function.^37^ Ongoing preclinical studies in LDL receptor null mice suggest that inhibiting ANGPTL3 suppresses LDL-C independently of the LDL receptor. Consistent with this, when patients with null-null homozygous familial hypercholesterolemia were treated with evinacumab, they observed substantial reductions in LDL-C.^36^

Because the effect on LDL-C with evinacumab is independent of LDL receptors, this therapeutic option may be particularly helpful in patients with LDL receptor mutations. Treatment options for these patients are limited.^43^ It was previously shown in a Phase 2 trial that evinacumab reduced LDL-C by 49% after 4 weeks of treatment among homozygous familial hypercholesterolemia patients.^36^ Patients with biallelic null LDL receptor mutations had a 34% drop in LDL-C on evinacumab.^36^ Evinacumab could potentially be considered for patients unable to reach LDL-C targets, such as those with severe heterozygous familial hypercholesterolemia, despite multiple lipid-lowering drugs or nephrotic syndrome; likewise, it could be used to help those who cannot tolerate other lipid-lowering drugs.

HDL-C reductions in both the single and multiple ascending dose studies resembled those of homozygous familial hypercholesterolemia patients treated with evinacumab.^36^ Lowered HDL-C probably stems from enhanced endothelial lipase activity. The clinical significance of the drop in HDL-C remains unclear. Although patients with inherently low HDL-C are more prone to atherosclerotic cardiovascular disease, it is not known whether lowering HDL-C in the context of suppressing the atherogenic lipids would predict atherosclerotic cardiovascular disease; to the contrary, low HDL-C in this context might well predict *lower* risk because it correlates with lower non–HDL-C, LDL-C, and apolipoprotein B.^44^ Along these lines, individuals with *ANGPTL3* LOF mutations have panhypolipidemia with low HDL-C and have a lower risk of coronary arterial disease.^45^ Preclinical studies and human genetic analyses suggest that lowering non–HDL-C, apolipoprotein B, and LDL-C via ANGPTL3 inhibition could, in turn, lower risk for cardiovascular events.^37^

Evinacumab was well tolerated, with no dose-related adverse events in the single dose study, and no dose-limiting toxicity identified during each dose escalation in the multiple ascending dose study. Elevations in liver function parameters in the single ascending dose study were from single elevations and not dose-related. Elevations in alanine aminotransferase and aspartate aminotransferase were not observed in the multiple ascending dose study. Naturally, further study will be needed to more fully characterize the adverse events.

As a limitation, our results are from Phase 1 studies with small sample sizes, where the studied population may not always overlap with the eventual target population of evinacumab. Other caveats include absence of study subjects with extreme hypertriglyceridemia or extreme hypercholesterolemia. The lipid profile of the subjects enrolled in this study had lipid elevations of much lower degree than the target hyperlipidemic patients requiring treatment with lipid-altering therapies. However, it should be noted that the effect of a single dose of evinacumab treatment in those with triglycerides ≥450 mg/dL or ≥1000 mg/dL will be reported in a separate manuscript. In the single ascending dose study, prospective subjects with an indication for statin therapy were excluded (Table I in the online-only Data Supplement, Exclusion 1). In the multiple ascending dose study, use of lipid-altering medications was exclusionary (Table II in the online-only Data Supplement, Exclusion 5). Moreover, we did not measure postprandial triglycerides (which are essential in ANGPTL3 metabolism) in these studies. There were a number of patient withdrawals and dropouts reported in both the studies; however, as described in the sample size section of the statistical analysis, this study was overenrolled to mitigate the number of dropouts and met the n=6 per group to adequately assess safety. Additionally, monoclonal antibody studies are generally longer than small molecule studies, making subject retention challenging. Although no drug toxicity concerns were reported, larger studies in patients with more substantial lipid disease, including patients treated with other lipid-lowering therapies, will be required to confirm the efficacy and safety of evinacumab.

In conclusion, ANGPTL3 inhibition with evinacumab was well-tolerated and lowered triglycerides by up to 80% within a few days in patients with elevated triglycerides. Treatment with evinacumab could be a strategy for those with severely elevated triglycerides.

## Acknowledgments

Data were collected at the study sites and were analyzed by representatives of Regeneron Pharmaceuticals Inc. The first draft of the manuscript was jointly written by Drs Ahmad and Dunbar, with review and revision by the other authors. The academic authors vouch for the accuracy and completeness of the data and analyses as presented and for the fidelity of this report to the trial protocol. We thank the patients, their families, and all investigators involved in this study. Medical writing assistance and editorial support were provided by Sophie K. Rushton-Smith, PhD, of MedLink Healthcare Communications Ltd, (London), as well as Grace Shim, PhD, and Aparna Shetty, PhD, of Prime (Knutsford, United Kingdom), all funded by Regeneron Pharmaceuticals Inc, according to Good Publication Practice guidelines. The authors were responsible for all content and editorial decisions, and received no honoraria related to the development of this publication.

## Sources of Funding

Both studies were sponsored by Regeneron Pharmaceuticals Inc.

## Disclosures

Dr Ahmad reports grants from Regeneron Pharmaceuticals Inc during the conduct of the study, personal fees from Regeneron Pharmaceuticals Inc, personal fees from Amgen, personal fees from Akcea, grants from the National Institutes of Health–National Heart, Lung, and Blood Institute, and grants from the Familial Hypercholesterolemia Foundation, outside the submitted work. Drs Hamon, Chan, Pordy, Mellis, Dansky, Banerjee, and Gipe are employees and shareholders of Regeneron Pharmaceuticals Inc. A. Bouzelmat and Dr Sasiela were employees and shareholders of Regeneron Pharmaceuticals Inc at the time of these studies. Dr Dunbar reports grants and nonfinancial support from Regeneron Pharmaceuticals Inc during the conduct of the study, as well as grants from Ionis, UniQure, Akcea, Amarin, and Astra-Zeneca outside the submitted work. He was employed by ICON Clinical Services during part of the manuscript preparation, and by Amarin for a brief portion, where he will also own stock.

## Supplementary Material
