# Supplementary material for: Inhibition of Angiopoietin-Like Protein 3 With a Monoclonal Antibody Reduces Triglycerides in Hypertriglyceridemia
Source: Circulation. 2019 Jun 27;140(6):470–86. doi: 10.1161/CIRCULATIONAHA.118.039107 (PMC6686956; doi:10.1161/CIRCULATIONAHA.118.039107)
Supplement: Supplementary file 1 [file cir-140-470-s001.pdf]

Supplemental Appendix

Supplemental Text 1. Additional Methods for the Single Ascending Dose Study

Sample Size Estimation

A sample size of 6 or more treated subjects at each dose level was considered adequate to evaluate the safety of a given dose. For subjects with elevations of triglycerides and/or low-density lipoprotein cholesterol (LDL-C), enrollment of 6 subjects at each evinacumab dose level and 10 placebo-treated subjects across all dose levels would provide 53% power to detect a treatment difference of 25% (standard deviation [SD] 22%) versus placebo on a percentage change from baseline in LDL-C at day 8 of the study when comparing each dose with placebo using a 2-sided test with a 0.05 significance level. The power would become 81% if the treatment difference was 30% (SD 19%). The power calculation also applies to triglycerides. All placebo-treated subjects were pooled regardless of their dose level.

Dose Escalation and Stopping Rules

Escalation to the next dose level occurred once 8 subjects enrolled in a dose level completed day 15 safety assessments and the data had been reviewed at a Dose Escalation Review meeting.

Dose Escalation Review

The Dose Escalation Review meeting analyzed the ongoing data from preceding dosing sequences based on the number of observed dose-limiting toxicities (DLTs) compared to the prespecified thresholds or an observed grade 4 serious adverse event (SAE) to inform whether dosing could proceed as planned in subsequent sequence or to stop further dose escalation. DLT was defined as: (1) any treatment-

emergent adverse event (TEAE) of grade 3 or greater toxicity as per Food and Drug Administration guidelines,[1] whether treatment-related or not (TEAEs that can clearly be ascribed to causes other than evinacumab were not considered to be DLTs); (2) any physical or laboratory finding that triggers diagnostic evaluation and therapeutic intervention by a medical subspecialist; (3) any grade 3 or greater systemic injection reaction (as defined by the National Cancer Institute Common Terminology Criteria for Adverse Events version 4.03 grading scale, for cytokine release syndrome).

The criteria for analyzing DLTs were as follows. (1) If DLTs were ≤1 in a dosing sequence within a single cohort, or ≤2 DLTs occurred in a dosing sequence within 2 cohorts, subjects in the next dosing sequence were randomized to receive either placebo or the planned dose of evinacumab. (2) If DLTs were ≥2 in a dosing sequence within a single cohort, or ≥3 DLTs occurred in a dosing sequence within 2 cohorts, dosing in the current sequence was suspended until a safety review has been conducted at a special Dose Escalation Review meeting. The outcome of the safety review decided whether to continue the study as planned, with re-evaluation if another DLT was observed at the current dosing sequence, or to stop dosing at the current dosing sequence and route (subcutaneous or intravenous). In the latter case, the observed toxicity was considered dose limiting. (3) If additional DLTs were observed such that criterion 2 was met in any dosing sequence, dosing in that sequence and any higher sequence was suspended until a special safety review meeting had been conducted to assess/determine the continuation of dosing in the suspended dosing sequence(s). (4) If a single grade 4 SAE was observed in any dosing sequence, dosing in the study was suspended and a comprehensive safety review was conducted prior to consideration of further dosing.

Supplemental Text 2. Additional Methods for the Multiple Ascending Dose Study

Sample Size Estimation

A sample size of up to 48 subjects, with 8 subjects in each of the 6 dose cohorts, was considered adequate to evaluate the safety of a given dose. Thirty-six of these subjects (6 in each cohort) were randomized to receive evinacumab and 12 (2 in each cohort) to receive placebo.

Study Cohorts and Dose Escalation

The study population was divided into 6 cohorts:

- Cohort 1 – evinacumab 150 mg SC QW × 8 doses or placebo.
- Cohort 2 – evinacumab 300 mg SC Q2W × 4 doses or placebo.
- Cohort 3 – evinacumab 300 mg SC QW × 8 doses or placebo.
- Cohort 4 – evinacumab 450 mg SC Q2W × 4 doses or placebo.
- Cohort 5 – evinacumab 450 mg SC QW × 8 doses or placebo.
- Cohort 6 – evinacumab 20 mg/kg IV Q4W × 2 doses or placebo.

Dosing was initiated at the following sequence:

- Dosing sequence 1: Cohorts 1 and 2 in parallel.
- Dosing sequence 2: Cohorts 3 and 4 in parallel.
- Dosing sequence 3: Cohort 5.
- Dosing sequence 4: Cohort 6.

Dose escalation was initiated after day 15 safety data from a current dosing sequence had been reviewed. Ongoing data from preceding dosing sequences were used to inform whether dosing could proceed as planned in subsequent sequence.

References

1. US Food and Drug Administration. Guidance for industry: Toxicity grading scale for healthy adult and adolescent volunteers enrolled in preventive vaccine clinical trials. Available from: https://www.fda.gov/downloads/BiologicsBloodVaccines/ucm091977. [Accessed November 29, 2018].

Supplemental Table 1. Study Criteria for Subjects with Mixed Dyslipidemia (Single Ascending Dose Study)

**Inclusion criteria**
1) Healthy men and women ages, 18–65 years, inclusive.
2) Subjects must have either a or b or both:
a) LDL-C ≥100 mg/dL at screening. A retest is permitted for subjects with LDL-C ≥95 mg/dL. The criteria will be based on directly measured LDL-C.
b) Serum TGs measured after a 12-hour fast ≥150 mg/dL. TGs will be measured twice, at least 4 days apart during the screening period. Both measurements must be ≥140 mg/dL and at least 1 must be ≥150 mg/dL.
3) Body mass index 18.0–35.0 kg/m^2.
4) Normal fasting blood glucose level (<100 mg/dL) at screening. A repeat fasting blood glucose test may be performed if the initial screening value is 100–105 mg/dL.
5) Standard 12-lead electrocardiogram after 10 minutes resting in a supine position; 120 ms < PR <220 ms, QRS <120 ms, QTc (Fridericia) ≤430 ms (males), 450 ms (females).
6) Willing to refrain from the consumption of more than 2 standard alcoholic drinks in any 24-hour period for the duration of the study. A standard alcoholic drink is the equivalent of 12 ounces of beer, 5 ounces of wine, or 1.5 ounces of hard liquor.
7) Willing to refrain from the consumption of alcohol for 24 hours prior to each study visit.
8) Willing to consistently maintain his/her usual diet for the duration of the study.
9) Willing to refrain from strenuous exercise for the duration of the trial.
10) Willing and able to comply with clinic visits and study-related procedures.
11) Provide signed informed consent.
12) For sexually active men (contraception is not required for men with documented vasectomy) and women of childbearing potential (postmenopausal women must be amenorrheic for at least 12 months in order not to be considered of childbearing potential. In cases where the duration of amenorrhea is uncertain, it is up to the discretion of the investigator to determine whether pregnancy testing should be performed. Pregnancy testing and contraception are not required for women with documented hysterectomy or bilateral tubal ligation or bilateral oophorectomy. In cases where documentation of hysterectomy, bilateral tubal ligation, or bilateral oophorectomy is not provided, the site should perform pregnancy testing), willingness to utilize adequate contraception and not become pregnant (or have their partner[s] become pregnant) for the full duration of the study. Adequate contraceptive measures include intrauterine device; bilateral tubal ligation; vasectomy; condom or diaphragm plus contraceptive sponge, foam, or jelly.
**Exclusion criteria**
1) Subjects indicated for the use of statins according to criteria in Adult Treatment Panel III Guidelines, as updated in 2004.

2) History of abnormal fasting blood glucose (≥100 mg/dL) or diabetes. One repeat measurement is allowed if the initial value falls between 100 and 105, inclusive
3) Initiation of a new exercise routine or major change to a previous exercise routine within 4 weeks prior to screening.
4) Initiation of a new diet or major change to a previous diet within 4 weeks prior to screening.
5) Use of a medication or nutraceutical that alters serum lipids within 4 weeks prior to screening, including but not limited to statins, ezetimibe, fibrates, niacin, omega-3 fatty acids, or bile acid resins.
6) Use of any concomitant medications, including prescription medications (including birth control pills), nutritional supplements, and over-the-counter medications (except for the occasional use of acetaminophen, aspirin, or nonsteroidal anti-inflammatory drugs), within 3 weeks of the day –1 visit (excluding medications that may be required for intercurrent illness).
7) Fasting serum TG >450 mg/dL measured after a 12-hour fast.
8) Creatine phosphokinase >3 x ULN at the screening visit (1 repeat lab is allowed).
9) Aspartate aminotransferase or alanine aminotransferase >2 x ULN at the screening visit (1 repeat lab is allowed).
10) Thyroid stimulating hormone level >1.5 x ULN or < lower limit of normal.
11) Glomerular filtration rate ≤70 mL/min (Cockcroft-Gault equation).
12) Any clinically significant abnormalities observed during the screening visit.
13) History of drug or alcohol abuse, or cigarette smoking within 1 year of screening.
14) Receipt of another investigational drug or therapy within 30 days or at least 5 half-lives (whichever is longer) prior to the screening visit.
15) Blood donation of any volume within 1 month prior to administration of study drug.
16) Receipt of any medication within 5 times the elimination or PD half-life of that drug prior to administration of evinacumab.
17) Significant concomitant illness or history of significant illness such as cardiac, renal, neurologic, endocrinologic, metabolic or lymphatic disease, or any other illness or condition that may adversely affect the subject’s participation in this study.
18) Known history of human immunodeficiency virus; and/or positive hepatitis B surface antigen, and/or positive hepatitis C antibody at the screening visit.
19) Hospitalization for any reason within 60 days of screening.
20) Previous exposure to any biological therapeutic.
21) History of a hypersensitivity reaction to doxycycline or similar compound.
22) History or presence of malignancy within 5 years prior to the screening visit (other than successfully treated non-metastatic cutaneous squamous cell or basal cell carcinoma, and/or localized carcinoma in situ of the cervix).

23)	Any medical or psychiatric condition which, in the opinion of the investigator, would place the subject at risk, interfere with participation in the study or interfere with the interpretation of the study results.
24)	Any subject who is the investigator or any sub-investigator, research assistant, pharmacist, study coordinator, or other staff directly involved in the conduct of the protocol, or family member of staff involved in the conduct of the protocol.
25)	Pregnant or breast-feeding women.

LDL-C, low-density lipoprotein cholesterol; TG, triglyceride; ULN, upper limit of normal.

Supplemental Table 2. Study Criteria for Subjects With Elevated Triglycerides and LDL-C (Multiple Ascending Dose Study)

**Inclusion criteria**
1) Provide signed informed consent.
2) Otherwise healthy men, and women who are not of childbearing potential (see exclusion criterion #29 for definition); age s18–65 years, inclusive.
3) Subjects must have both a and b:<br>a) Serum LDL-C ≥100 mg/dL at screening. A retest is permitted for subjects with LDL-C ≥95 mg/dL.<br>b) Serum TG ≥150 mg/dL, measured after an 8-hour fast. Triglycerides will be measured twice, at least 4 days apart, during the screening period. Both measurements must be ≥140 mg/dL, and at least 1 measurement must be ≥150 mg/dL.
4) Body mass index 18.0 kg/m^2 to 38.0 kg/m^2, inclusive.
5) Vital signs at screening after resting in sitting position for 5 minutes:<br>• 95 mmHg < systolic blood pressure ≤140 mmHg.<br>• 60 mmHg < diastolic blood pressure ≤90 mmHg.<br>• 45 bpm < heart rate <100 bpm.
6) Blood pressure controlled (systolic blood pressure ≤140 mmHg, diastolic blood pressure ≤90 mmHg) on no more than 2 antihypertensive medications.
7) Standard 12-lead electrocardiogram after 10 minutes resting in a supine position;120 ms < PR <220 ms, QRS <120 ms, QTc (Fridericia) <430 ms (males) or <450 ms (females).
8) Willing to refrain from the consumption of more than 2 standard alcoholic drinks in any 24-hour period during the duration of the study. A standard alcoholic drink is the equivalent of 12 ounces of beer, 5 ounces of wine, or 1.5 ounces of hard liquor.
9) Willing to refrain from the consumption of alcohol for 24 hours prior to each study visit.
10) Willing to refrain from cigarette smoking for 24 hours prior to each study visit.
11) Willing to consistently maintain his/her usual diet for the duration of the study.
12) Willing to refrain from strenuous exercise for the duration of the trial.
13) Willing and able to comply with clinic visits and study-related procedures.
14) For sexually active men (contraception is not required for men with documented vasectomy; sperm donation is prohibited during and for up to 3 months after participation in the study), willingness to use 2 medically acceptable forms of birth control (medically acceptable contraceptive measures include vasectomy; condom; and contraceptive foam or jelly), should the subjects have sexual intercourse during their participation in this research study. Men are required to use double-barrier contraception (condom with spermicide) from study start until 3 months after the last study visit.

Exclusion criteria
1) Subjects with newly diagnosed diabetes mellitus (within 3 months of screening), or who are poorly controlled (HbA1c >8.5%) at the full screening visit.
2) Current insulin use.
3) Current use of any medication, other than metformin, that alters blood glucose control.<br>• Chronic use of metformin in subjects with type 2 diabetes is permitted in the study, provided the dose has been stable within 3 months prior to screening and is to remain stable for the duration of the study.
4) Use of any concomitant medications, including prescription medications (including birth control pills; except stable use of antihypertensives), nutritional supplements, and over-the-counter medications (except for metformin, and the occasional use of acetaminophen, aspirin, or non-steroidal anti-inflammatory drugs), within 3 weeks of the day –1 visit (excluding medications that may be required for intercurrent illness).<br>• Low-dose aspirin (<100 mg/day) is permitted, provided it remains at a stable dose throughout the study).<br>• Up to 2 antihypertensives are permitted (except for hydralazine), provided they remain at a stable dose throughout the study.
5) Use of a medication or nutraceutical that alters serum lipids within 4 weeks prior to screening, including, but not limited to, statins, ezetimibe, niacin, omega-3 fatty acids, bile acid resins, red yeast rice, or fibrates (within 6 weeks prior to screening).
6) Initiation of a new exercise routine or major change to a previous exercise routine within 4 weeks prior to screening.
7) Initiation of a new diet or major change to a previous diet within 4 weeks prior to screening.
8) Fasting serum TGs >500 mg/dL measured after an 8-hour fast.
9) Creatine phosphokinase >3 × ULN at the screening visit (1 repeat lab is allowed).
10) Aspartate aminotransferase or alanine aminotransferase >2 × ULN at the screening visit (1 repeat lab is allowed).
11) Thyroid stimulating hormone >1.5 × ULN or < lower limit of normal.
12) Calculated creatinine clearance <70 mL/min (Cockcroft-Gault equation).
13) Any clinically significant abnormalities observed during the screening visit.
14) History of drug and/or alcohol abuse within 1 year of screening.
15) Exposure to another investigational drug or therapy within 30 days, or within at least 5 half-lives (whichever is longer) prior to the screening visit.
16) Blood donation of any volume within 1 month prior to administration of study drug.
17) Significant concomitant illness or history of significant illness, such as cardiac (including documented coronary heart disease), renal, neurologic, endocrine (except adequately controlled type 2 diabetes with HbA1c ≤8.5%), metabolic, or lymphatic disease, or any other illness or condition that may adversely affect the subject’s participation in this study.

18)	Class II, III, or IV congestive heart failure defined by the New York Heart Association Classification.
19)	History of cerebrovascular accident, transient ischemic attack, coronary bypass graft surgery, percutaneous coronary intervention, carotid surgery or stenting, unstable angina pectoris, or myocardial infarction within 6 months before the screening visit.
20)	Known history of human immunodeficiency virus infection; and/or positive hepatitis B surface antigen, and/or positive hepatitis C antibody at the screening visit.
21)	Hospitalization for any reason within 60 days of screening.
22)	Previous adverse reaction to any investigational or therapeutic biological agent.
23)	History of a hypersensitivity reaction to doxycycline or similar compound.
24)	Platelet count <100,000/mm^3 at the screening visit.
25)	History or presence of malignancy within 5 years prior to the screening visit (other than successfully treated non-metastatic cutaneous squamous cell or basal cell carcinoma, and/or localized carcinoma in situ of the cervix).
26)	Any medical or psychiatric condition which, in the opinion of the investigator, would place the subject at risk, interfere with participation in the study, or interfere with the interpretation of the study results.
27)	Any subject who is the investigator or any sub-investigator, research assistant, pharmacist, study coordinator, or other staff directly involved in the conduct of the protocol, or family member of staff involved in the conduct of the protocol.
28)	Pregnant or breast-feeding women.
29)	Women of childbearing potential (in order not to be considered of childbearing potential, female patients require clinical confirmation of postmenopausal status (at least 12 months since last menses without an alternative medical cause; confirmed by postmenopausal levels of follicle stimulating hormone >20 mIU/mL) or permanently sterile (does not include tubal ligation).

HbA1c, glycated hemoglobin; LDL-C, low-density lipoprotein cholesterol; TG, triglyceride; ULN, upper limit of normal.

Supplemental Table 3: Pharmacokinetic Parameters of Total Serum Evinacumab Concentrations

		Total Evinacumab											
		Single Ascending Dose Study											
		5 mg/kg IV				10 mg/kg IV				20 mg/kg IV			
**Parameter**	**Units**	**N**	**Mean**	**Median**	**SD**	**N**	**Mean**	**Median**	**SD**	**N**	**Mean**	**Median**	**SD**
t_{max}	day	10	0.0724	0.0660	0.0333	9	0.0836	0.0833	0.0290	11	0.108	0.0840	0.0575
C_{max}	mg/L	10	177	179	23.0	9	313	326	43.0	11	591	555	71.9
C_{max}/dose	kg/L	10	35.3	35.7	4.59	9	31.3	32.6	4.30	11	29.6	27.8	3.60
AUC_{last}	day* mg/L	10	1093	1020	170	9	3261	2991	775	11	7706	7715	1512
AUC_{last}/ dose	day*kg/L	10	219	204	34.1	9	326	299	77.5	11	385	386	75.6
CL	L/day /kg	10	0.00466	0.00490	0.000620	9	0.00320	0.00334	0.000774	10	0.00259	0.00256	0.000448
		75 mg SC				150 mg SC				250 mg SC			
		**N**	**Mean**	**Median**	**SD**	**N**	**Mean**	**Median**	**SD**	**N**	**Mean**	**Median**	**SD**
t_{max}	day	11	2.27	2.00	0.786	12	2.67	3.00	0.492	9	4.22	3.00	2.10
C_{max}	mg/L	11	1.89	1.05	1.98	12	7.88	5.67	6.23	9	18.8	18.4	8.46
C_{max}/dose	1/L	11	0.0252	0.0140	0.0264	12	0.0525	0.0378	0.0416	9	0.0752	0.0736	0.0338

AUC_{last}	day* mg/L	11	9.66	7.01	10.2	12	62.8	39.4	52.5	9	167	165	120
AUC_{last}/ dose	day/L	11	0.129	0.0935	0.135	12	0.419	0.263	0.350	9	0.667	0.661	0.480
CL/F	L/day	7	7.48	6.05	6.38	12	5.95	3.73	6.93	7	6.17	1.26	13.3
		**Multiple Ascending Dose Study**											
		**150 mg QW SC**				**300 mg Q2W SC**				**300 mg QW SC**			
		**N**	**Mean**	**Median**	**SD**	**N**	**Mean**	**Median**	**SD**	**N**	**Mean**	**Median**	**SD**
t_{max}	day	6	2.60	3.17	1.98	6	15.6	13.8	6.56	6	14.6	14.6	4.66
C_{max}	mg/L	6	0.0173	0.0211	0.0132	6	0.0520	0.0458	0.0219	6	0.0485	0.0487	0.0155
C_{max}/dose	1/L	6	2.17	1.50	2.48	6	4.50	4.50	2.74	6	4.50	4.50	2.74
AUC_{last}	day* mg/L	6	995	1052	573	6	749	515	746	6	4535	4836	2191
AUC_{last}/ dose	day/L	6	6.63	7.02	3.82	6	2.50	1.72	2.49	6	15.1	16.1	7.30
AUC_{tau}	day* mg/L	6	13.8	15.9	11.5	5	125	88.9	68.4	6	75.9	73.6	32.1
CLss/F	L/day	5	23.7	8.09	35.0	5	2.86	3.38	1.09	6	4.97	4.08	3.20
		**450 mg Q2W SC**				**450 mg QW SC**				**20 mg/kg Q4W IV**			
		**N**	**Mean**	**N**	**Mean**	**N**	**Mean**	**N**	**Mean**	**N**	**Mean**	**Median**	**SD**
t_{max}	day	7	24.1	7	24.1	7	24.1	7	24.1	7	602	596	58.5
C_{max}	mg/L	7	0.0535	7	0.0535	7	0.0535	7	0.0535	7	30.1	29.8	2.92

C_{max}/dose	1/L	7	7.00	7	7.00	7	7.00	7	7.00	7	0.0774	0.0833	0.0288
AUC_{last}	day* mg/L	7	2565	7	2565	7	2565	7	2565	7	18329	19655	8859
AUC_{last}/ dose	day/L	7	5.70	7	5.70	7	5.70	7	5.70	7	916	983	443
AUC_{tau}	day* mg/L	7	257	7	257	7	257	7	257	6	6535	6525	682
CLss/F	L/day	7	2.25	7	2.25	7	2.25	7	2.25	6	3.09	3.07	0.326

AUC, area under the concentration-time curve; AUC_{last}, AUC computed from the time zero to the time of the last positive concentration; AUC_{tau}, AUC during a dosing interval; CLss/F, apparent clearance at steady state; C_{max}, peak concentration; CL, total body clearance; CL/F, apparent clearance; CLss, clearance at steady state; IV, intravenous; N, number of subjects; NA, not applicable; SC, subcutaneous; SD, standard deviation; t_{max}, time to Cmax; QW, once a week; Q2W, once every 2 weeks.

Note: C_{max}, C_{max}/dose, and t_{max} for multiple dosing study are reported for the first dose; AUCs are reported for the last dose.

Supplemental Table 4. Changes Over Time in Liver Function Test Parameters (IU/L) in Patients with Elevations in Alanine Aminotransferase (Single Ascending Dose Study)

Study day	Patient 1 (SC 250 mg)	Patient 2 (IV 5 mg/kg)	Patient 3 (IV 10 mg/kg)	Patient 4 (IV 10 mg/kg)	Patient 5 (IV 10 mg/kg)	Patient 6 (IV 10 mg/kg)	Patient 7 (IV 20 mg/kg)
Baseline	25	16	72	43	35	81	65
Change from baseline							
Day 1, hour 8	6	3	0	−4	5	5	−5
Day 2	14	9	13	−1	23	20	−12
Day 3	77*	26	49	6	36	38	−6
Day 4	96*	48	42	11	47	58*	−3
Day 8	35	29	−13	39	15	39	2
Day 11	16	25	−7	53	6	9	−13
Day 15	23	5	−22	28	−1	−13	3
Day 22	3	2	−24	14	−3	−28	25
Day 29	6	0	−27	5	0	−28	−11
Day 43	−4	1	−40	10	−2	−38	0
Day 64	−1	NR	NR	−17	−8	−38	7
Day 85	NR	NR	NR	−5	20	−39	−8

End of study[†]	21	2	−38	−8	−6	−19	−1

*>3 ULN and baseline CPK ≤3 ULN.

[†]Day 106 was the end of study visit for the first 4 dose levels (75, 150, and 250 mg SC and 5 mg/kg IV); day 126 was the end of study visit for the fifth and sixth dose levels (10 and 20 mg/kg IV).

CPK, creatine phosphokinase; IV, intravenous; NR, not reported; SC, subcutaneous; ULN, upper limit of normal.

Supplemental Table 5. Changes Over Time in Liver Function Test Parameters (IU/L) in Patients with Elevations in Aspartate Aminotransferase (Single Ascending Dose Study)

Study day	Patient 1 (SC 250 mg)	Patient 2 (IV 5 mg/kg)	Patient 3 (IV 10 mg/kg)	Patient 4 (IV 10 mg/kg)
Baseline	23	21	40	35
Change from baseline				
Day 1, hour 8	6	2	1	1
Day 2	11	7	14	8
Day 3	63	21	30	11
Day 4	52	31	17	20
Day 8	9	10	−1	12
Day 11	1	10	−3	13
Day 15	4	2	−10	−2
Day 22	2	3	−10	1
Day 29	7	1	−5	−6
Day 43	−2	3	−11	−10
Day 64	−1	NR	NR	−8
Day 85	NR	NR	NR	−9

End of study*	11	5	−11	−6

*Day 106 was the end of study visit for the first 4 dose levels (75, 150, and 250 mg SC and 5 mg/kg IV); Day 126 was the end of study visit for the fifth and sixth dose levels (10 and 20 mg/kg IV).

IV, intravenous; NR, not reported; SC, subcutaneous.

Supplemental Table 6. Changes Over Time in Liver Function Test Parameters (IU/L) in Patients with Elevations in Creatine Phosphokinase (Single Ascending Dose Study)

Study day	Patient 1 (SC 75 mg)	Patient 2 (SC 150 mg)	Patient 3 (SC placebo)	Patient 4 (SC 150 mg)	Patient 5 (SC 250 mg)	Patient 6 (IV 5 mg/kg)	Patient 7 (IV placebo)	Patient 8 (IV 10 mg/kg)	Patient 9 (IV placebo)
Baseline	139	247	144	121	89	206	366	103	625
Change from baseline									
Day 1, Hour 8	−29	−45	1	−39	−15	−55	48	−1	−218
Day 2	−31	−42	−26	−49	−22	−37	18	−1	−273
Day 3	−31	37	−18	−33	−28	−23	19	7	−297
Day 4	−30	57	−18	−33	−32	−27	−29	8	−303
Day 8	83	6	78	638†	18	81	71	217	39
Day 11	56	933*	193	270	−6	1	257*	721*	277*
Day 15	60	510*	13	77	−10	70	315*	131	216
Day 22	40	322	48	350	1151*	164	74	531*	570*
Day 29	−37	228	345	188	47	12	164	129	421*
Day 43	1546*	231	113	105	17	123	112	54	511*
Day 64	−8	350	212	111	45	179	−24	134	649*

Day 85	23	1033†	300	29	45	456†	44	83	458*
End of study†	69	361	476*	51	47	56	67	79	701*

*>3 ULN and baseline CPK ≤3 ULN.

†Day 106 was the end of study visit for the first 4 dose levels (75, 150, and 250 mg SC, and 5 mg/kg IV); Day 126 was the end of study visit for the fifth and sixth dose levels (10 and 20 mg/kg IV).

CPK, creatine phosphokinase; IV, intravenous; SC, subcutaneous; ULN, upper limit of normal.

Supplemental Table 7. Changes Over Time in Liver Function Test Parameters (IU/L) in Patients with Elevations in Alanine Aminotransferase (Multiple Ascending Dose Study)

Study day	Patient 1<br>(Placebo)
Baseline	21
Change from baseline	
Day 6	6
Day 7	18
Day 9	43
Day 13	0
Day 14	−2
Day 15	−10
Day 16	8
Day 17	−7
Day 18	−2
Day 19	12

Supplemental Table 8. Changes Over Time in Liver Function Test Parameters (IU/L) in Patients with Elevations in Aspartate Aminotransferase (Multiple Ascending Dose Study)

Study day	Patient 1 (Placebo)
Baseline	24
Change from baseline	
Day 6	−3
Day 7	17
Day 9	26
Day 13	−4
Day 14	−5
Day 15	−9
Day 16	1
Day 17	−5
Day 18	2
Day 19	25

Supplemental Table 9. Changes Over Time in Liver Function Test Parameters (IU/L) in Patients with Elevations in Creatine Phosphokinase (Multiple Ascending Dose Study)

Study day	Patient 1<br>(20 mg/kg Q4W)
Baseline	98
Change from baseline	
Day 6	25
Day 7	36
Day 9	5
Day 13	18
Day 14	4
Day 15	1319*
Day 16	3
Day 17	−2
Day 18	7
Day 19	4

*>3 and ≤10 ULN, and CPK ≤3 ULN, at baseline.

CPK, creatine phosphokinase; Q4W, every 4 weeks; ULN, upper limit of normal.

Supplemental Figure 1. Study Flow Diagram for Subjects With Mixed Dyslipidemia (A) Single Ascending Dose Study (B) Multiple Ascending Dose Study

(A)

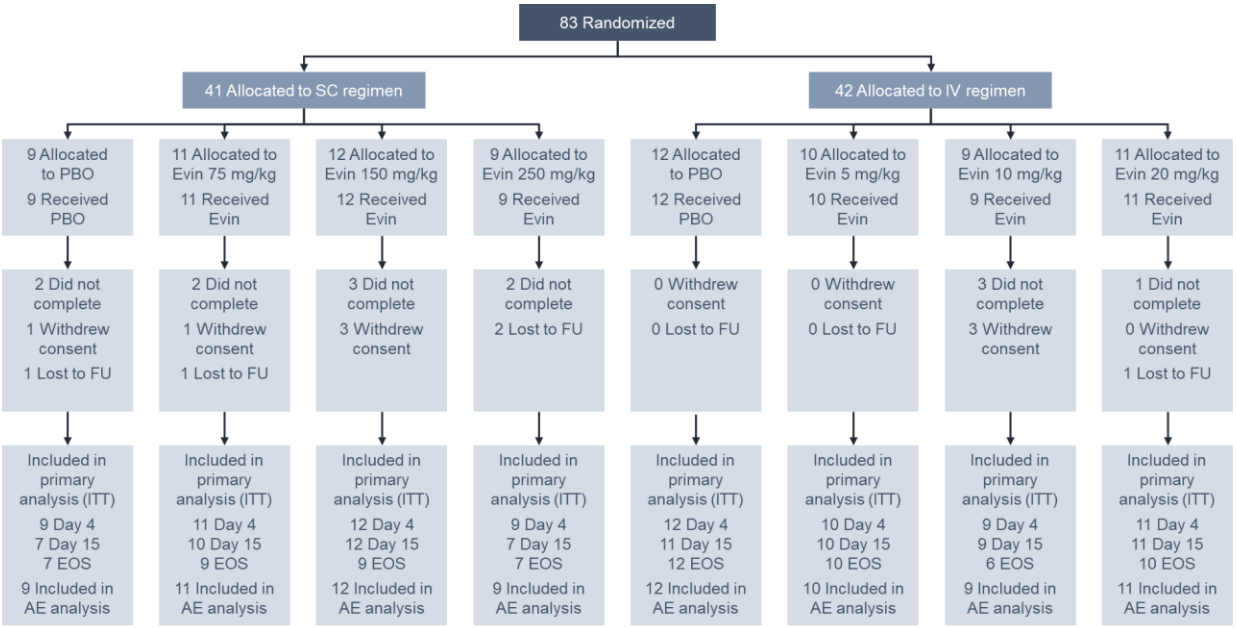

59 subjects were enrolled under the original protocol, 8 under amendment 1, and 16 under amendment 2.

(B)

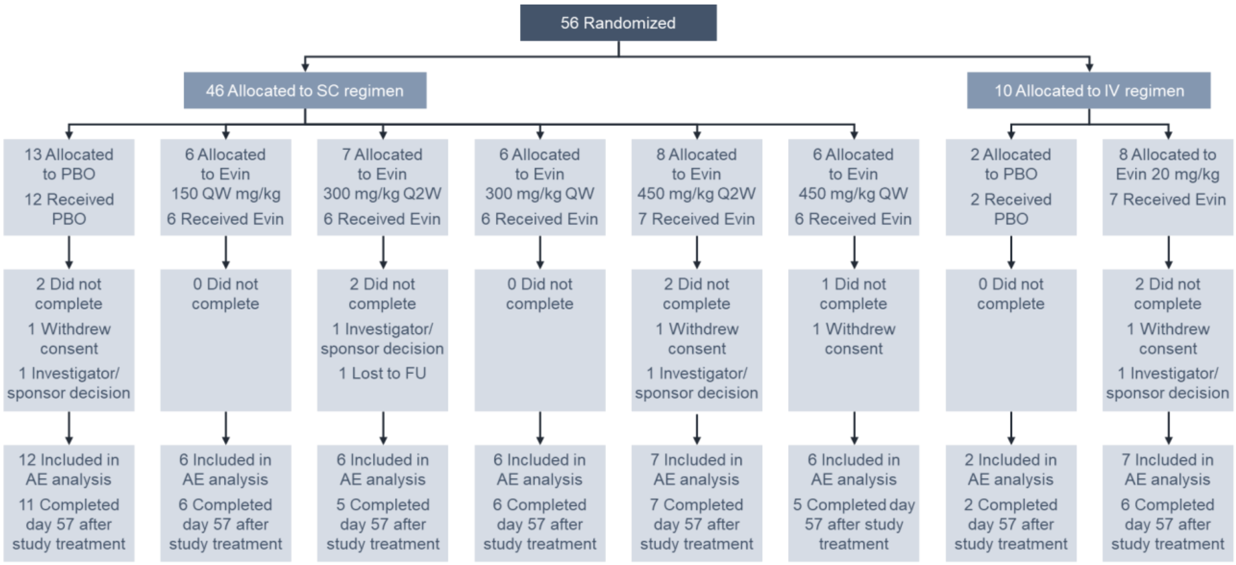

AE, adverse event; EOS, end of study; Evin, evinacumab; FU, follow-up; ITT, intention-to-treat; IV, intravenous; PBO, placebo; SC, subcutaneous. Q2W, every 2 weeks; QW, every week

Supplemental Figure 2. Mean (±SE) Concentrations of Total Serum ANGPTL3 in the (A) Single Ascending Dose and (B) Multiple Ascending Dose Studies

(A)

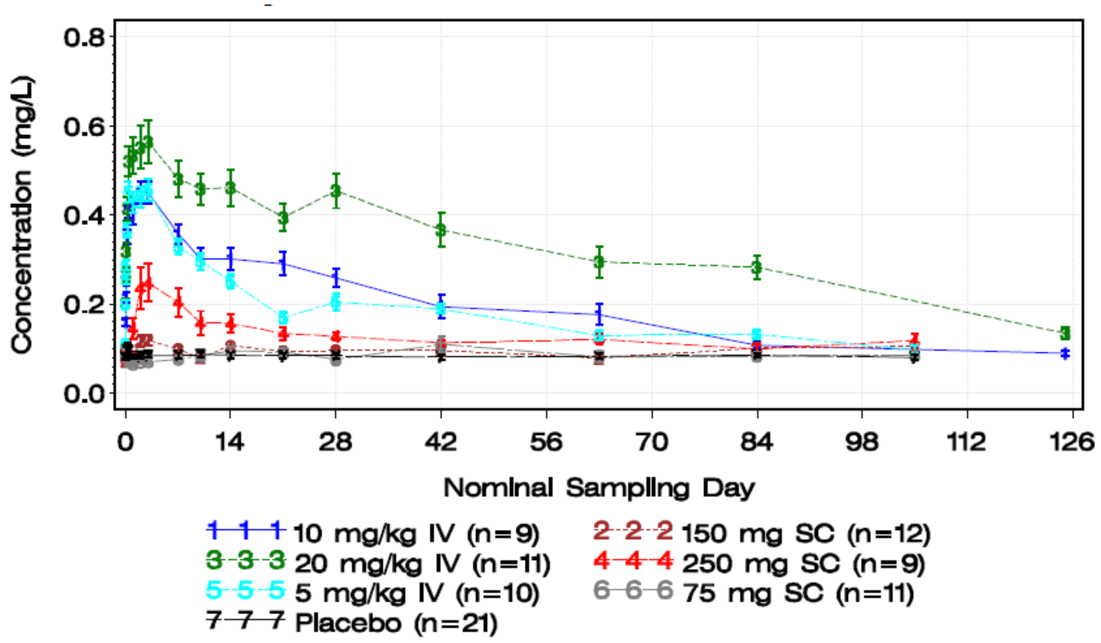

(B)

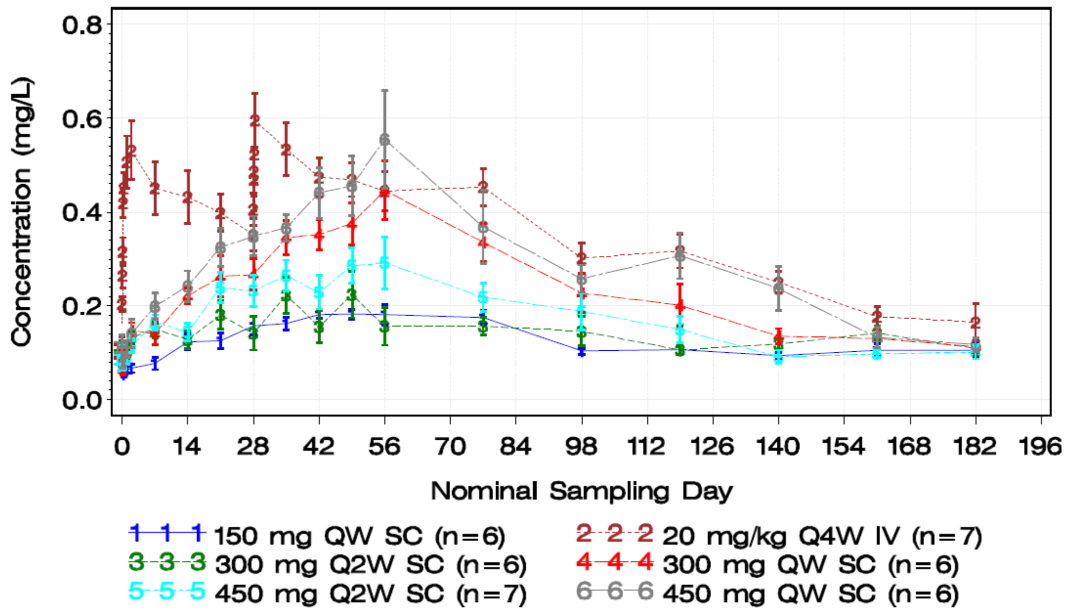

Supplemental Figure 3. Percent Change From Baseline in (A) Non-HDL-C and (B) Apolipoprotein B, and (C) Absolute Mean Apolipoprotein B Levels in Subjects With Multiple Dyslipidemia (Single Ascending Dose Study)

(A)

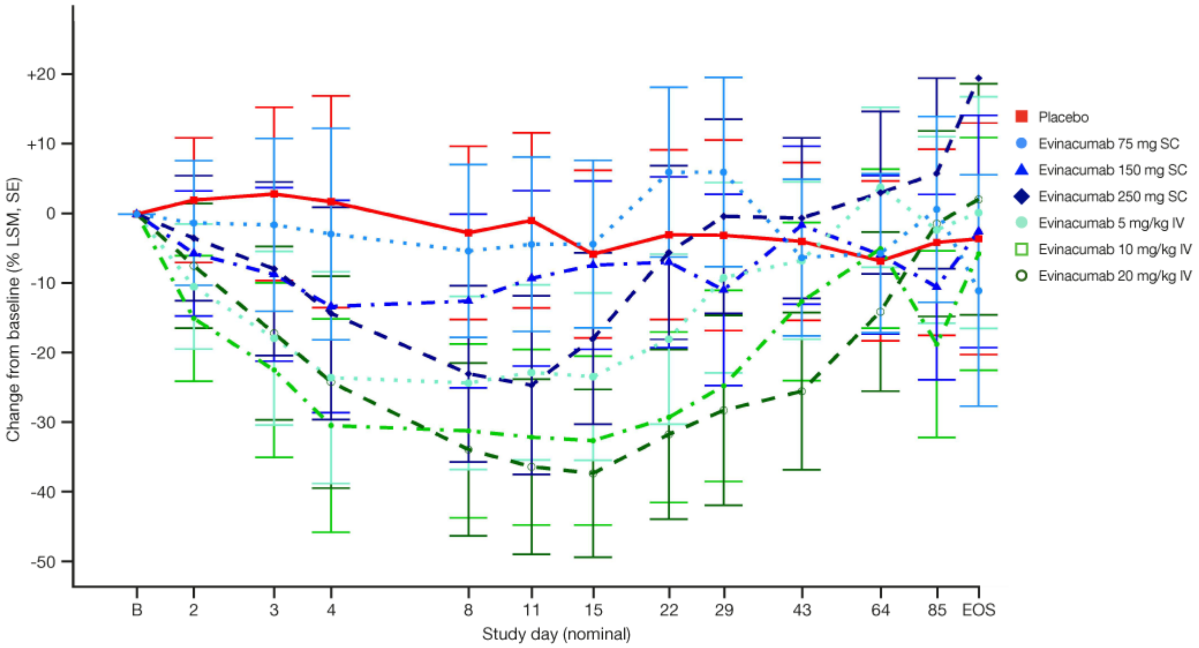

(B)

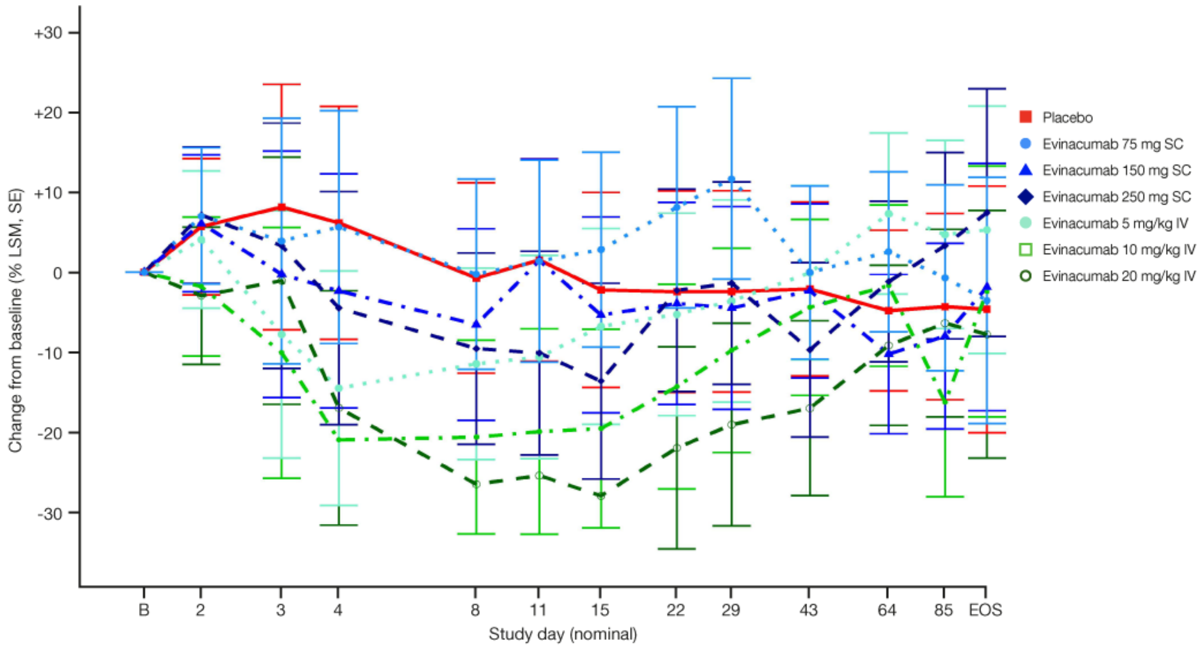
+30
+20
+10
0
-10
-20
-30
Change from baseline (% LSM, SE)
B
2
3
4
8
11
15
22
29
43
64
85
EOS
Study day (nominal)
Placebo
Evinacumab 75 mg SC
Evinacumab 150 mg SC
Evinacumab 250 mg SC
Evinacumab 5 mg/kg IV
Evinacumab 10 mg/kg IV
Evinacumab 20 mg/kg IV

(C)

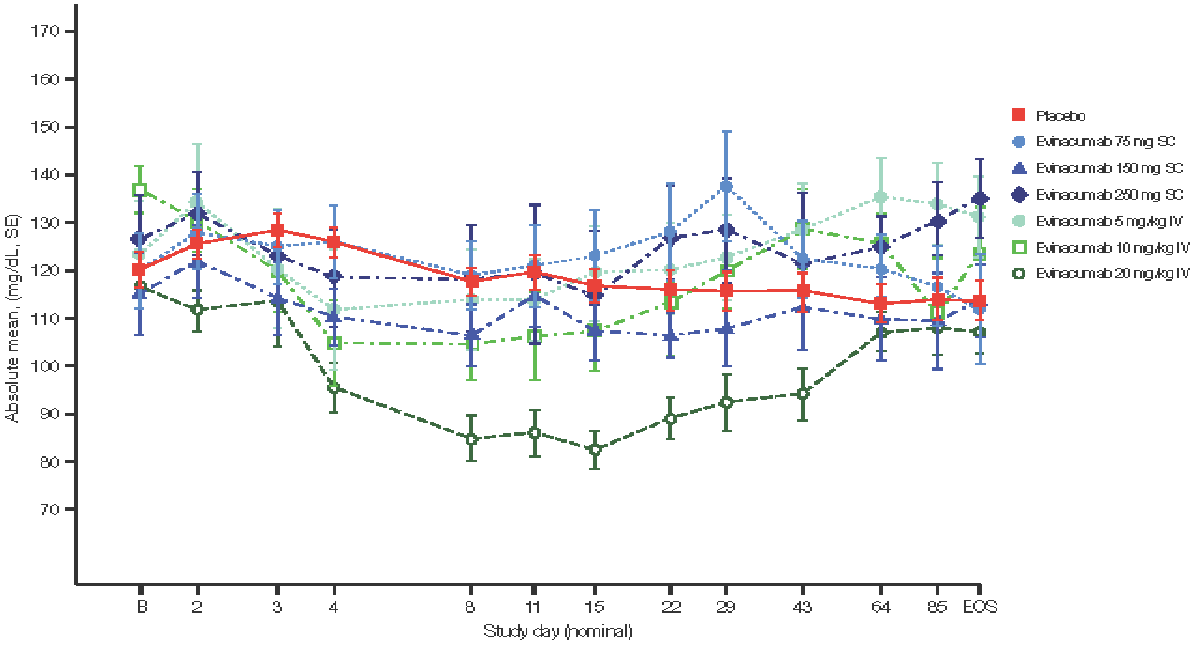

IV, intravenous; LSM, least-squares mean; SC, subcutaneous; SE, standard error.

Supplemental Figure 4. Percent Change From Baseline in (A) Non-HDL-C and (B) Apolipoprotein B and (C) Absolute Mean Apolipoprotein B Levels in Subjects With Elevated Triglycerides and LDL-C (Multiple Ascending Dose Study)

(A)

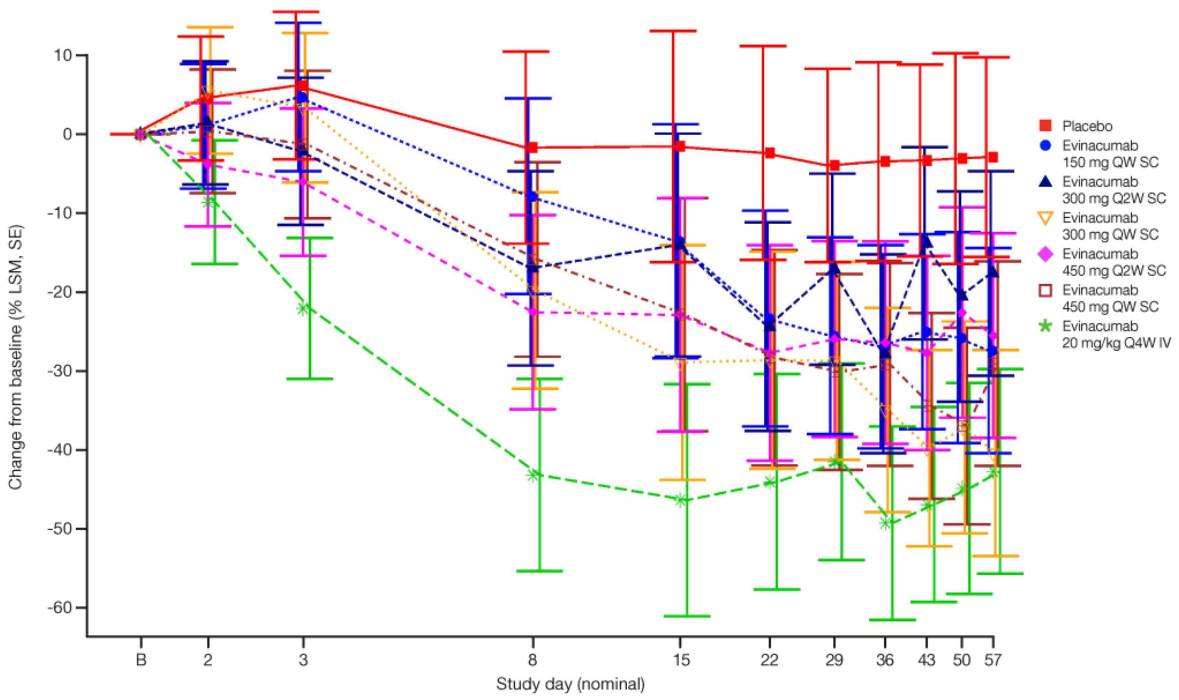

(B)

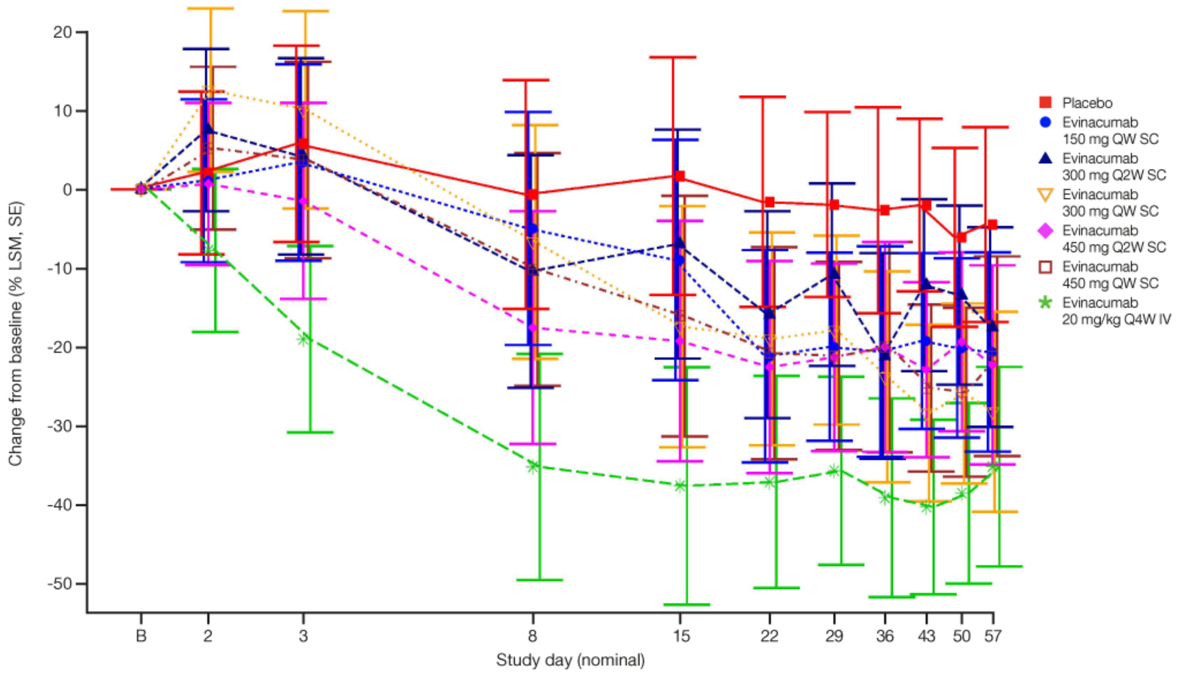

Change from baseline (% LSM, SE)
20
10
0
-10
-20
-30
-40
-50
B
2
3
8
15
22
29
36
43
50
57
Study day (nominal)
Placebo
Evinacumab 150 mg QW SC
Evinacumab 300 mg Q2W SC
Evinacumab 300 mg QW SC
Evinacumab 450 mg Q2W SC
Evinacumab 450 mg QW SC
Evinacumab 20 mg/kg Q4W IV

(C)

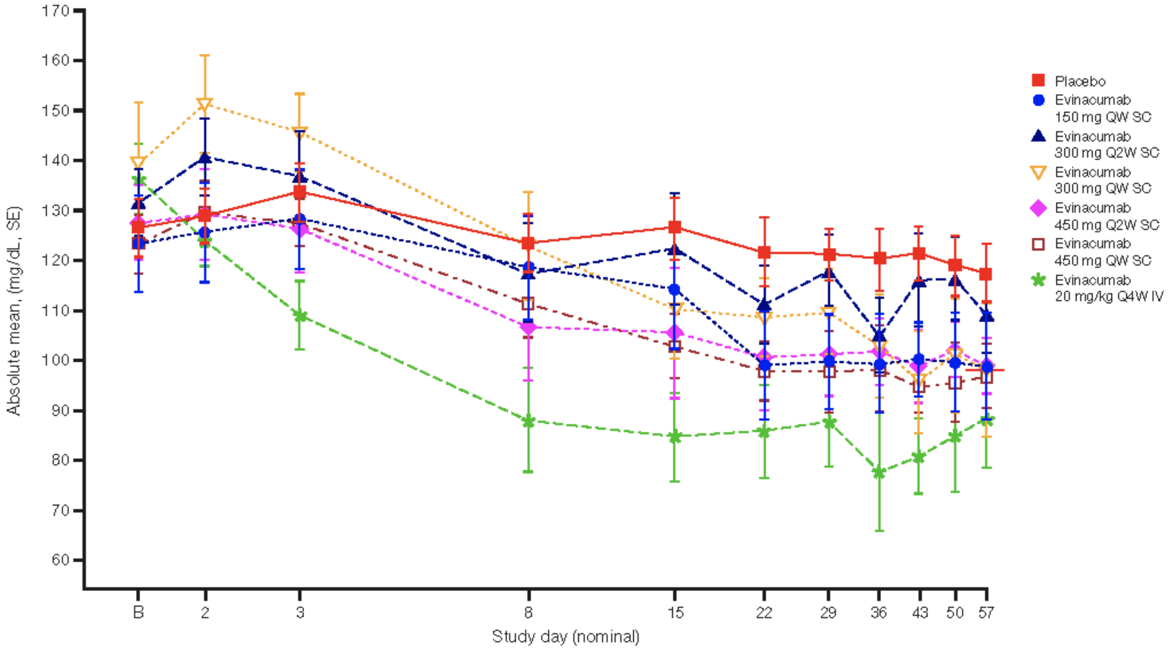

170
160
150
140
130
120
110
100
90
80
70
60
Absolute mean, (mg/dL, SE)
B
2
3
8
15
22
29
36
43
50
57
Study day (nominal)
Placebo
Evinacumab 150 mg QW SC
Evinacumab 300 mg Q2W SC
Evinacumab 300 mg QW SC
Evinacumab 450 mg Q2W SC
Evinacumab 450 mg QW SC
Evinacumab 20 mg/kg Q4W IV

Supplemental Figure 5. Percent Change From Baseline in (A) Lp(a), (B) Apolipoprotein A1, and (C) Total Cholesterol in Subjects With Multiple Dyslipidemia (Single Ascending Dose Study)

(A)

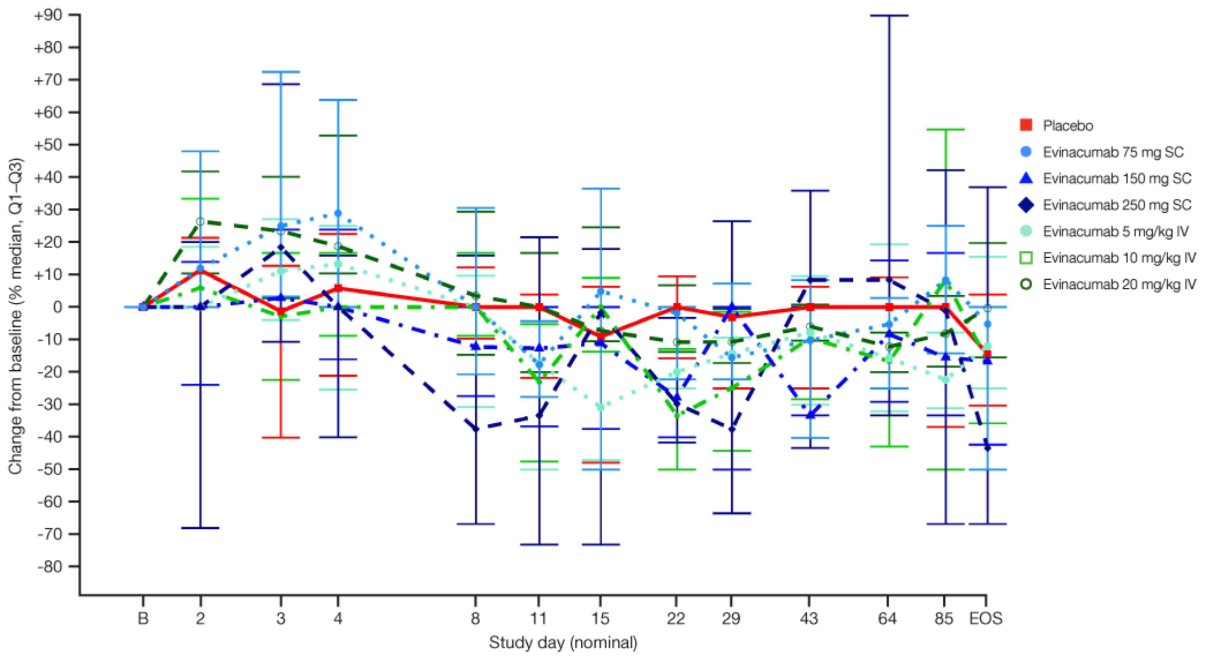

(B)

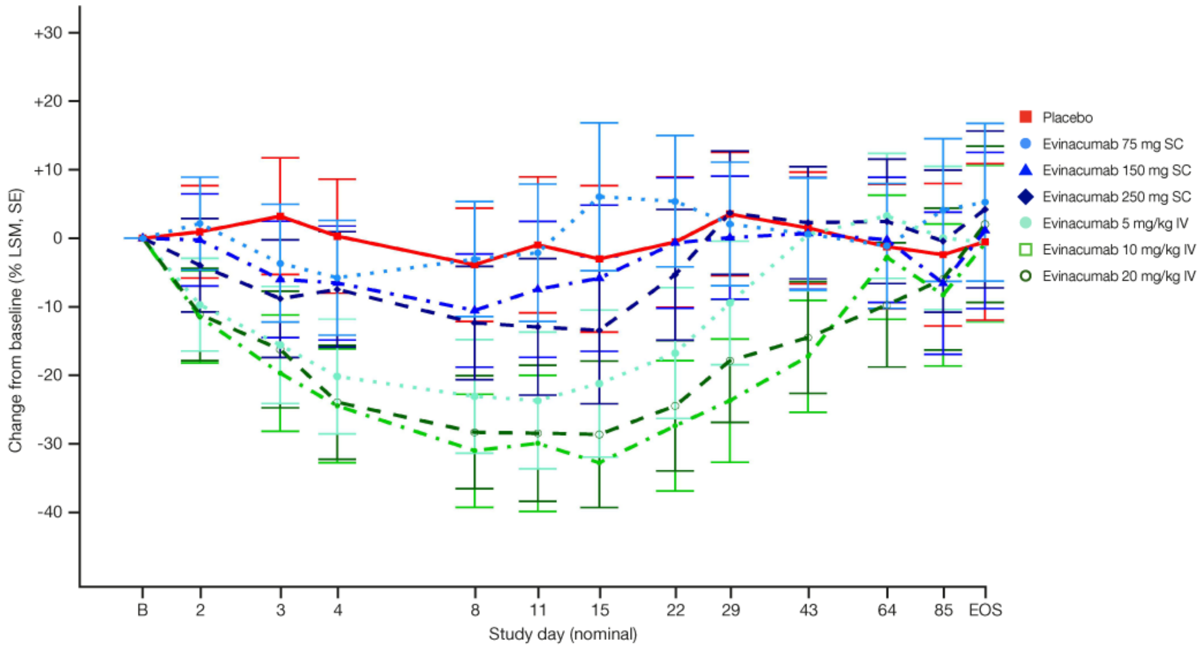

Change from baseline (% LSM, SE)
+30
+20
+10
0
-10
-20
-30
-40
B
2
3
4
8
11
15
22
29
43
64
85
EOS
Study day (nominal)
Placebo
Evinacumab 75 mg SC
Evinacumab 150 mg SC
Evinacumab 250 mg SC
Evinacumab 5 mg/kg IV
Evinacumab 10 mg/kg IV
Evinacumab 20 mg/kg IV

(C)

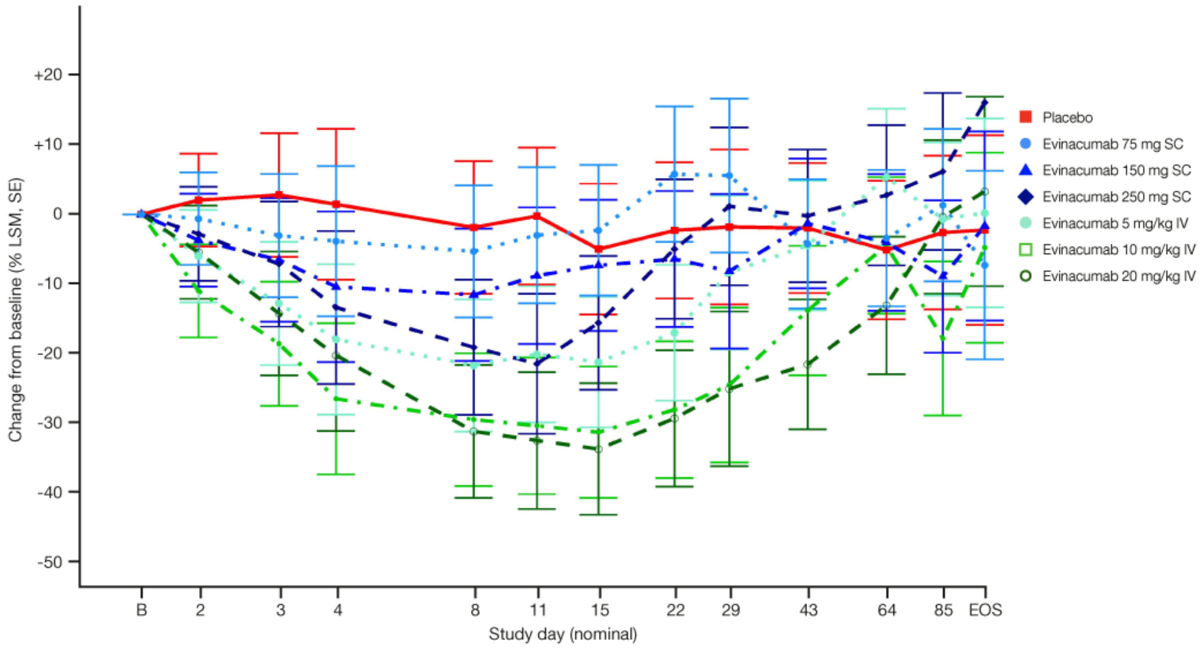

HDL-C, high-density lipoprotein cholesterol; IV, intravenous; Lp(a), lipoprotein (a); LSM, least-squares mean; SC, subcutaneous; SE, standard error.

Supplemental Figure 6. Percent Change From Baseline in (A) Lp(a), (B) Apolipoprotein A1 and (C) Total Cholesterol in Subjects With Elevated Triglycerides and LDL-C (Multiple Ascending Dose Study)

(A)

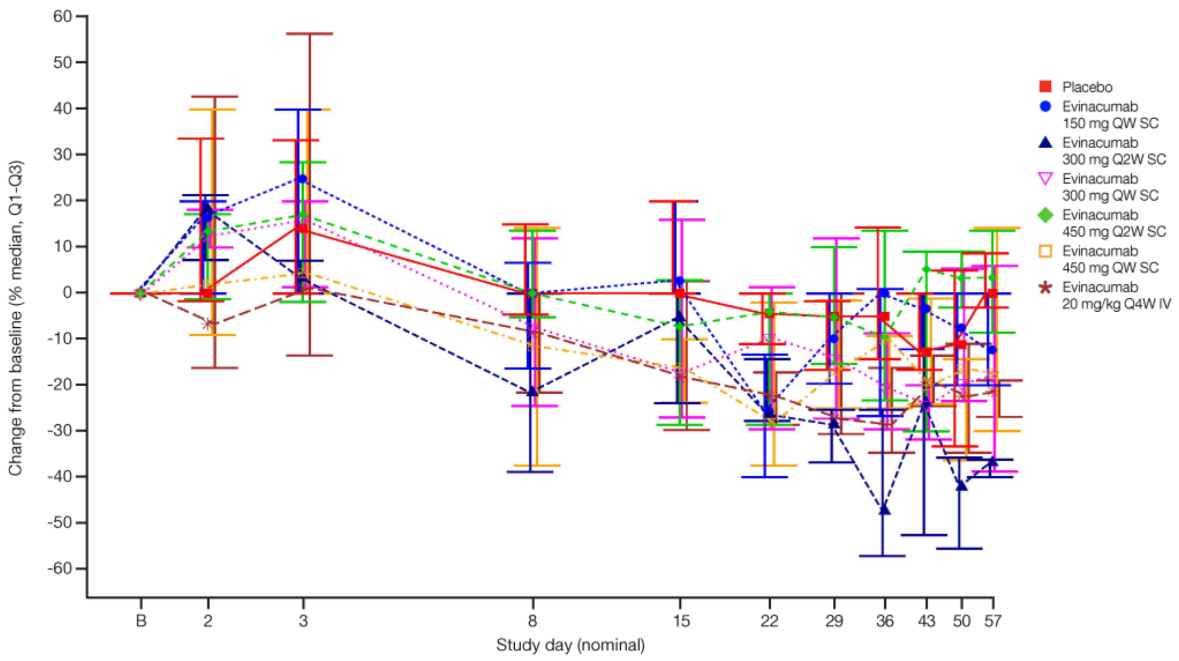

(B)

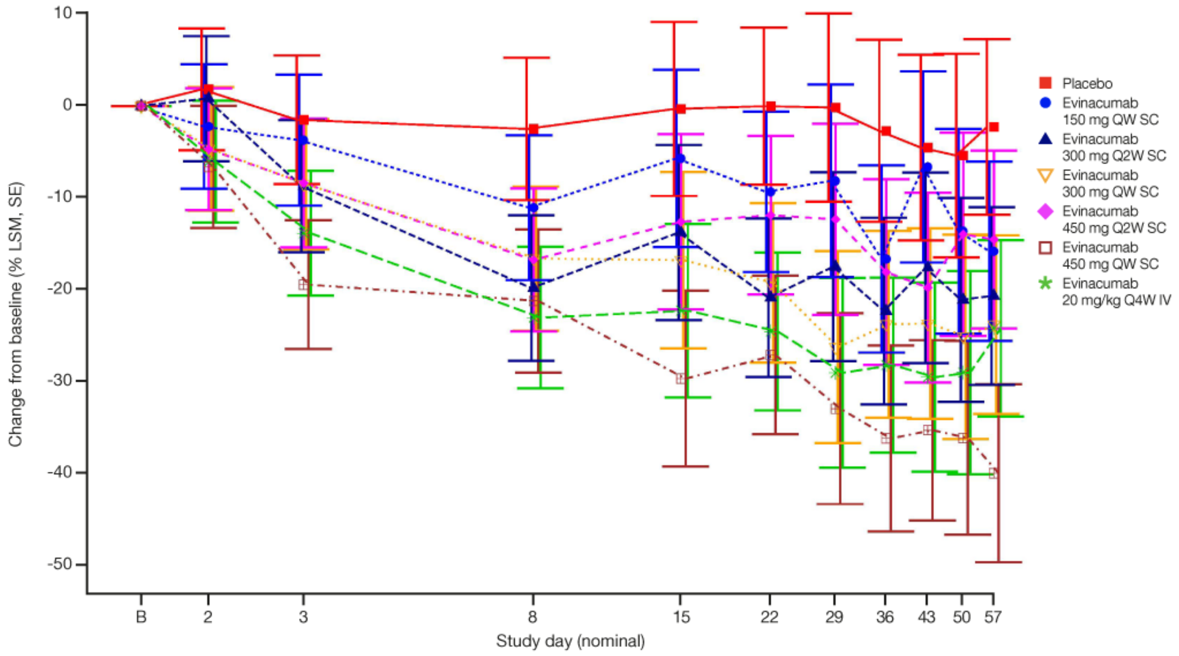

10
0
-10
-20
-30
-40
-50
Change from baseline (% LSM, SE)
B
2
3
8
15
22
29
36
43
50
57
Study day (nominal)
Placebo
Evinacumab 150 mg QW SC
Evinacumab 300 mg Q2W SC
Evinacumab 300 mg QW SC
Evinacumab 450 mg Q2W SC
Evinacumab 450 mg QW SC
Evinacumab 20 mg/kg Q4W IV

(C)

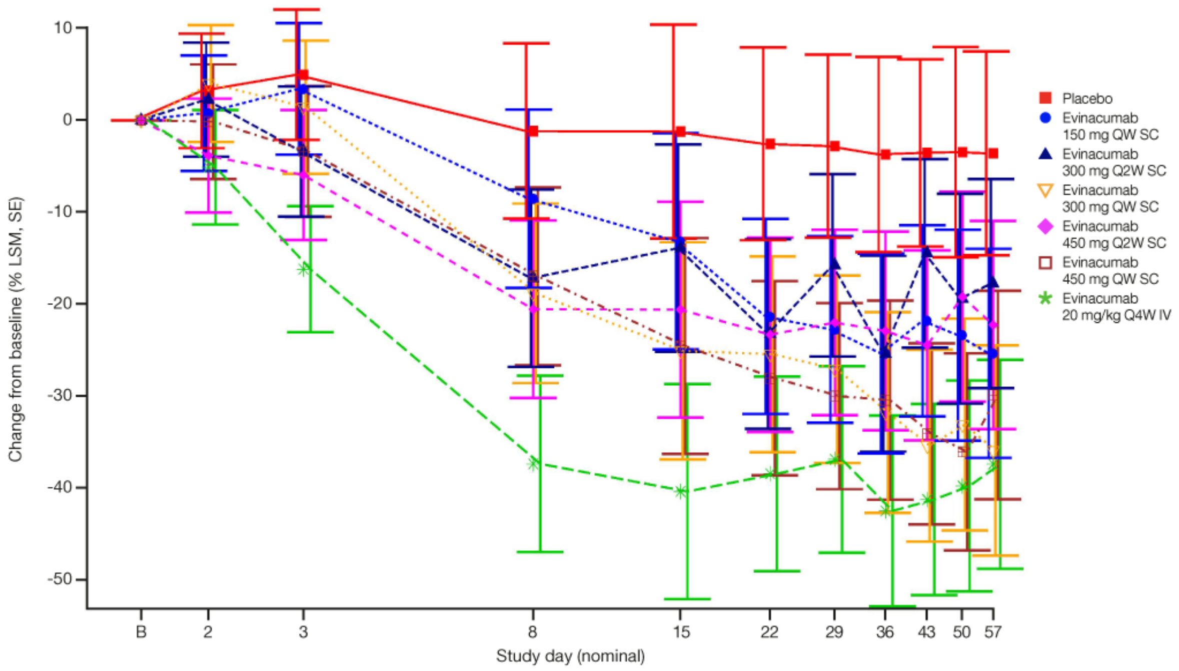

HDL-C, high-density lipoprotein cholesterol; IV, intravenous; Lp(a), lipoprotein (a); LSM, least-squares mean; Q2W, every 2 weeks; Q4W, every 4 weeks; QW, every week; SC, subcutaneous; SE, standard error.
